# Assessment for Sustainable Use of Quarry Fines as Pavement Construction Materials: Part I—Description of Basic Quarry Fine Properties

**DOI:** 10.3390/ma12081209

**Published:** 2019-04-12

**Authors:** Yinning Zhang, Leena Katariina Korkiala-Tanttu, Henry Gustavsson, Amandine Miksic

**Affiliations:** Department of Civil Engineering, Aalto University, 00076 Aalto, Finland; leena.korkiala-tanttu@aalto.fi (L.K-T.); henry.gustavsson@aalto.fi (H.G.); amandine.miksic@aalto.fi (A.M.)

**Keywords:** sustainable, secondary materials, quarry waste, frost susceptibility

## Abstract

As a secondary material, quarry fines are a valuable material to be reused for many purposes in civil engineering projects. The aggregate source depletion, especially the lack of high quality aggregates as expected in the future, as well as the demand for a carbon-neutral society and circular economy, also promotes the high-volume utilization of secondary materials such as quarry fines. The aim of this study is to do a feasibility assessment including a series of laboratory tests and analyses to evaluate the properties of quarry fine materials to determine if this type of material could be qualified as pavement construction material in high volume. The gradation information obtained from both sieving and hydrometer tests indicates the frost susceptibility of unstabilized quarry fines, therefore frost heave tests were performed and which further suggest the necessity of stabilization to improve its properties for pavement applications, especially in structural layers such as base, subbase, or filter layers. Some other general information and properties of unbound quarry fines, especially regarding their validity for application in pavement engineering are also investigated and discussed.

## 1. Introduction

One big challenge in road engineering is the economic and ecological use of soil and aggregate materials. The design of pavement layers in Finland is mainly based on empirical methods, which are presented as tabulated model structures [1]. This has led to a situation where high quality virgin aggregates have been used in excess and the use of secondary materials has been challenging [2]. Finland, in proportion to its population, is one of biggest user of aggregates inside the European Union. Annually, 80–100 million tons of aggregates are used in Finland [3]. The conditions of cold climate and long distances caused by the relatively low density of population both increase the need to use a lot of aggregates [4]. Road and street construction consumes about 50 million tons of non-renewable aggregates and the amount of CO_2_ emissions caused is 0.8 million tons annually [5]. Besides, in Europe the built environment is responsible for 40% of greenhouse gases emission, 50% of resource extraction, and 30%–45% of waste production [6]. According to Ehrukainen, the application of secondary or recycled materials was only 1% of the total use of aggregates [7]. Of the total consumption of aggregates, around 10% is used to produce concrete and 10% to produce asphalt concrete [4]. Therefore, it is of vital importance to significantly increase the utilization of “waste” materials.

Finland has a good source of natural rock for aggregate production, thus the motivation to use recycled material has been low in the past. Now, with the increase in the price of natural aggregates as well as the increased level of consciousness regarding environmental preservation and the promotion of a circular economy, strategies and targets have been proposed and are now enforced. The European Commission set four waste directives in 2018 [8]. The focus of the directives is to decrease the amount of waste, to increase recycling, and to diminish the need for landfills. A more solid aim is to increase the recycling, reuse, and other utilization of building and demolition waste up to 70% in weight by 2020 [9]. The United Nations Agenda for Sustainable Development, called Agenda 2030, also sets targets towards carbon-neutral societies by 2030 [10]. Finland has committed itself to taking action in regards to this agenda, including the aim to promote carbon-neutrality and the wise use of resources [11]. Many big cities, e.g., Helsinki, Espoo, Vantaa, Tampere, and Turku have defined their own targets for carbon neutrality and a circular economy. For example, the city of Helsinki has set a target to be carbon-neutral by 2035 [12]. These targets also require a much higher level of circular economy and promote the use of secondary materials. In summary, the reduction in natural resources and increasing consciousness regarding environment preservation have led to the established trend of utilizing secondary materials at higher levels in the near future, which is also the motivation of this study.

## 2. Literature Review

According to United States Environmental Protection Agency (EPA), non-hazardous secondary materials refers to any materials that are not the primary product of manufacturing or commercial processes but are the byproducts of such processes, including post-consumer material, post-industrial material, and scrap [13]. The European Commission also defines the waste that can be recycled and injected back into the economy as secondary raw materials [14]. On one hand, these materials may be considered as waste which needs to be treated and disposed of. On the other hand, many of them can have material or British Thermal Unit (BTU) value, with the possibility of being reused and managed for other industrial purposes with little or no harm to the environment.

Quarry waste is one such type of secondary material that unavoidably comes from the overburden, interburden, and extraction and processing of aggregates and includes quarry fines from processing activities [15]. As a general concept, quarry waste actually consists of different material types known as “quarry fines”, “quarry dust”, “stone byproducts”, “recycled aggregates”, “quarry powder wastes”, and so forth [16]. Quarry fines have become a real problem in the quarry industry because of two main reasons. Firstly, the higher the fraction of fines contained in the material, the more easily the material can be dislocated by gravity, wind, and water, and the more difficult the material is to handle, store, and dispose of. Very fine crusher dust can be generated during crushing and screening processes and is easily dispersed into the atmosphere, contaminating the air. Secondly, the recent tax policy has promoted the possible utilization or management of large stockpiles of quarry fines to minimize the adverse social and environmental effects caused by this material, but effective high-volume applications are underdeveloped. Moreover, many places around the world will be facing problems of reduced rock resources in the future, which will result in an inadequate production of aggregates and an increase in high quality aggregate prices. Quarry fines could provide alternatives to alleviate the problem of aggregate shortages in the civil engineering industry. Consequently, methods of utilization of quarry fines are in demand, regardless of the quarry sources.

Table 1 presents a substantial amount of under-utilized quarry fines produced and accumulated at Lemminkäinen Infra Oy in Finland over the recent years. Besides Lemminkäinen Infra Oy, there are some other aggregate producers. For example, Destia Oy had a total of 194 000 tons of quarry fines around Finland in 2015 [17]. These numbers indicate a clear potential for economic and environmental benefits from the appropriate utilization and application of this material.

Similarly to other granular unbound materials, one possible way of utilizing this type of quarry waste material is for landfill or pavement constructions. In Europe, the project “Alternative materials in road construction (ALT-MAT)” undertaken from 1998 to 1999 has been focused on utilizing different types of secondary/alternative materials for pavement base/subbase layers [19]. Many of the investigated secondary materials, including municipal solid waste incinerator (MSWI) bottom ashes and all types of slags, are fine-grained unbound materials that have shown positive results (both mechanically and environmentally) as pavement base/subbase materials. However, even though previous research has proven that there is a promising future for applications using many of the secondary materials in road construction, the research into quarry fines, and especially Finnish quarry fines, is still inadequate and many detailed aspects require further study. These aspects include physical and mechanical properties, durability evaluation, leaching properties, quarry fine applications in high volume, and so forth.

Since 2007, there has been an increase in research focusing on utilizing quarry waste as a construction material in the field of civil engineering. It has been implemented to fabricate artificial aggregates [20], hemp concrete [21], and mortars. In general, quarry fine materials have been found to be effective in improving the properties of conventional civil engineering materials, which has also been the most common application of this material in previous research. Quarry fines have been proven to be effective in mitigating the early decreasing of strength due to fly ash application [22]. It was also found that the partial replacement of cement by granite quarry dust is beneficial in several aspects including good durable behavior, less drying shrinkage and expansion, lower possibility of early-age cracking, while keeping strength properties comparable [23]. When used as stabilizer, quarry waste could improve the properties of soils in terms of better workability, a decrease in optimal water content and an increase in maximum dry unit weight [24,25], better swell and shrinkage properties for expansive soil [26], and satisfactory durability under freeze-thaw cycles [27]. Quarry fines were also employed as an addition to natural soils to improve properties such as grading and compaction characteristics, strength, and to reduce swelling and plasticity [24]. For flexible pavements, quarry fines have been utilized to improve physical, mechanical, and swelling properties of soil subgrade and subbase [28].

Extended research attempts have been made to utilize quarry waste in constructions for both non-bearing [21,29] and structural purposes [20,22,23,29,30,31]. Non-bearing blocks made of quarry waste and other additives are common for wall partitions and decorations. In addition, the most common application area of quarry fines in Finland today is as the uppermost unbound layer of yards, fields, and low-volume streets [17]. For structural purposes, there are mainly three types of effective applications utilizing quarry waste. One is to use quarry waste as a substitute for sands/filler in different percentages in concrete [22,30,32,33], and the other is to replace part of cement by the quarry waste to produce concrete [22], or even employ quarry waste as an additive to improve soil properties [23,24,25,26,27]. In other words, it is common to employ quarry waste as an alternative material to reduce the dosage of other materials (more expensive, more in demand, or less environmentally friendly) or as an additive, rather than using quarry waste as a major construction material. In fact, most of the previous research applies less than 50% quarry waste [19,20,21,23,24,25,26,27], and the majority applies 5% to 30% as aggregates/fillers to develop different types of concrete. This might be related to the high requirements for quality and the mechanical properties of concrete materials for adequate structural performance. Especially when applied to asphalt concrete for pavement constructions, quarry waste is only recommended by researchers for low-to-moderate volume traffic [32,34]. Another key point that can be summarized from current research is the inadequate durability evaluation of quarry-waste-based construction materials. There is only limited research conducted into durability evaluations of quarry-waste-based materials, and some of these have shown a reduction in durability with the incorporation of quarry waste [30]. However, a qualified pavement construction material should have enough durability in moist environments and also adequate frost-resistance for its application in cold regions. It should also be noted that the applications of the secondary quarry fine materials in ground water area would need permissions from the environment agencies in Finland, especially where active use of ground water is needed.

In this study, a comprehensive assessment including a series of laboratory tests and analyses was conducted to evaluate the properties of quarry fine materials, which have not been well-established in the past. Even though quarry fines have shown very promising results as an alternative or additive material in previous research, its properties as a pavement construction material are not clear. Unlike most previous studies, which apply quarry wastes only in a limited amount, the main purpose of this study was to determine if this type of byproduct could be qualified as a pavement base, subbase, or any other construction material, and explore a way to utilize it in high volumes. The benefits of utilizing this byproduct in high volumes include reducing the carbon footprint and promoting a circular economy, considering it can be easily found and obtained at most quarry sites. In addition, another inadequate aspect of previous research, the evaluation of the durability of quarry fines, was investigated here in term of a frost-susceptibility test to verify the suitability of this material for applications in cold regions. Some basic information and properties of unbound quarry fines, especially regarding their validity for application in pavement engineering were investigated and discussed here as a preliminary study and foundation to further research.

## 3. Material Sampling and Preparation

In general, adopting a proper sampling method using a specified apparatus helps avoid biased sampling and provides reliable test results. To obtain representative samples of the average properties of a batch at plants, the European standard SFS-EN 932-1 “Tests for general properties of aggregates. Part 1: Methods for sampling” has been followed for material sampling [35]. It should be noted that even though sampling from the stationary conveyor belt or from the stream of material is recommended, in practice, the quarry fines were only available from stockpiles at the plants when the authors collected them. However, to avoid segregation-caused biased results, sampling increments were randomly selected from as many parts of the batch as possible, and an adequate number of sampling increments was used to reduce the influence of the heterogeneity of the batch.

In total, around 100 kg of quarry fines with a 0–4 mm particle size were collected from the Koskenkylä quarry of Destia Oy, packaged into 10-kg capacity buckets, and directly transferred back to the laboratory for storage. The collected quarry fines are from high quality granite host rock according to CE marking, with major mineral compositions of plagioclase (50.4%) and quartz (39.6%). Reduction of a bulk sample by a riffle box or by the quartering method, as recommended in the standard, was conducted to obtain the amount of materials required for further research purposes. The reduced batches were heated in oven at (110 ± 5) °C to remove excess moisture and obtain a constant mass following the European standard SFS-EN 1097-5 “Tests for mechanical and physical properties of aggregates. Part 5: Determination of the mater content by drying in a ventilated oven” [36]. This was important to maintain the identical status of all of the batches so that water content can be accurately controlled for any further specimen preparation and testing.

## 4. Description of Basic Quarry Fine Properties

### 4.1. Gradation of the Quarry Fines

The particle size distribution of the quarry fines with a 0–4mm grain size was obtained according to European standard SFS-EN 933-1 “Tests for geometrical properties of aggregates. Part 1: Determination of particle size distribution. Sieving method” [37]. The wet sieving method is necessary for naturally agglomerated materials or materials containing a large amount of fine fractions because fine fractions with static charges are more prone to clumping. Washing breaks down the agglomerations and also helps the separation of fines from coarse fractions [38], which improves the results of particle distribution from the successive sieving process.

#### 4.1.1. Wet Sieving Method

**Figure 1:**
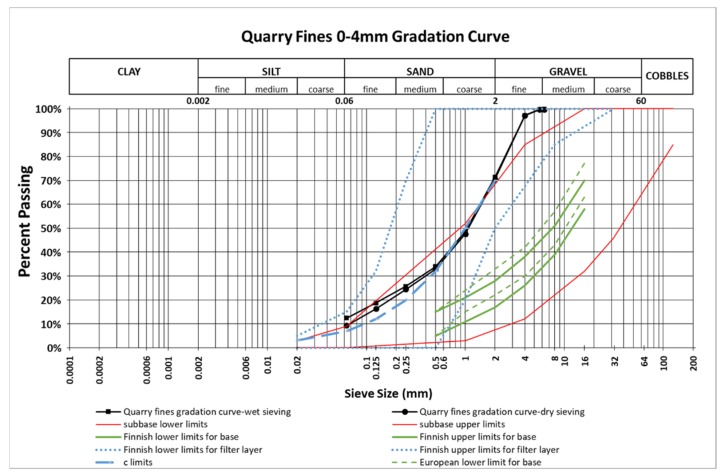
Gradation curves of quarry fines with a 0–4 mm particle size and the limits for pavement base, subbase, and filter layers according to Finnish and European requirements.

A trial test using the wet sieving method was conducted to determine the fine contents of the quarry fines. It showed that the fine content of quarry fines reaches as high as 12.5%. According to the trial test results, it was decided that the wet sieving method and a hydrometer test are necessary for the quarry fines in order to get reliable results due to the large amount of fine fractions (<0.063 mm). Figure 1 presents the gradation curve of quarry fines with a 0–4 mm particle size obtained from both the wet and dry sieving methods, together with the limits for pavement base, subbase, and filter layers required by Finnish and European standards (Tables S1 and S2). The gradation curve of quarry fine materials as obtained from wet and dry sieving methods shows differences in finer grains but the difference appears to have no significant effects regarding the qualification for base/subbase/filter layer requirements. It was found that the quarry fines are not qualified for pavement base materials according to the gradation information only, whereas around half of the gradation curve stay inside the limits for subbase [39]. However, because of the gradation curve that sits on the lower limit of subbase requirements, it is hard to validate quarry fines for either base or subbase from the gradation information only, and thus a mechanical test to determine the strength of quarry fines will be planned and performed in the future. As a filter layer, the material can be qualified completely since the gradation curve sits inside the limits, stays only at one side of the c limit, and also satisfies the requirement *D*_15_/ *d*_85_ < 5 [39].

*D*_15_ is a grain size equivalent to 15 percent of the permeation of granular material (insulating layer material); *d*_85_ is the grain size of the finer material (bottom), having a permeation percentage of 85.

The coefficient of uniformity (*C*_u_) and the coefficient of gradation (*C*_c_) are calculated by Equations (1) and (2) to evaluate the grade of quarry fines. The classifications as listed in the European standard SFS-EN ISO 14688-2:2018 “Geotechnical investigation and testing” [40]. Identification and classification of soil. Part 2: Principles for a classification” were followed to categorize the grading of quarry fines used in this study.

(1) $$C_{u}=\frac{D_{60}}{D_{10}}$$

(2) $$C_{c}=\frac{{\left(D_{30}\right)}^{2}}{D_{10}\times D_{60}}$$

For well-graded sand, $C_{u}>15$ and $1\leq C_{c}\leq 3$.

**Figure 2:**
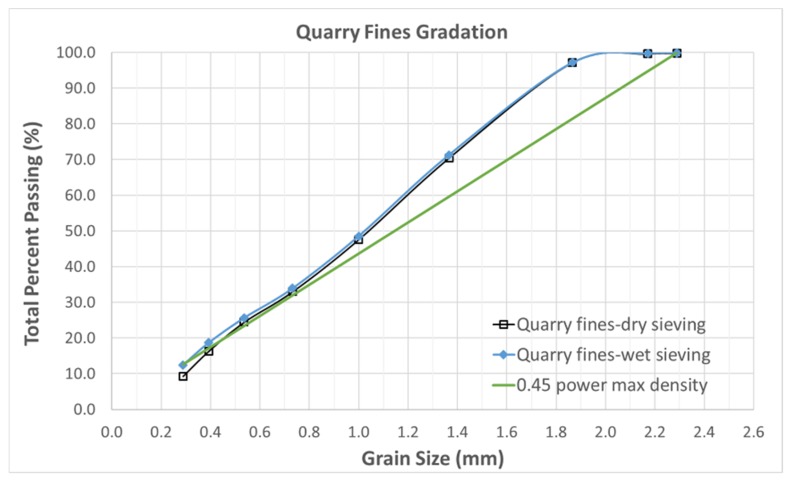
0.45 power maximum density graph for quarry fines.

The coefficient of uniformity (*C*_u_) and the coefficient of gradation (*C*_c_) were determined to be 28.8 and 2.2, respectively. Therefore, the quarry fines can be classified as well-graded materials. The same classification is also valid according to the Unified Soil Classification System. From the 0.45 power maximum density graph shown in Figure 2, it can be observed that the quarry fines have the gradation characteristics of both fine- and dense-graded material, with the fine-graded characteristic being dominant. This characteristic is better captured when a wet sieving method is applied.

#### 4.1.2. Hydrometer Method

The particle size distribution of the fine fraction significantly influences the frost susceptibility of granular materials, which can be determined by hydrometer test. A quarry fine sample containing only fine fraction (finer than 0.063 mm) was obtained by sieving the 0–4 mm quarry fine material mechanically and was tested for gradation with the hydrometer test. This method is based on the phenomenon of particles of different sizes settling by gravitation in a liquid at different rates, as well as Strokes’ law, which establishes a relationship between the terminal velocity of a particle and other parameters of the fluid and material properties. The European standard SFS-EN ISO 17892-4: 2016 “Geotechnical investigation and testing” [41]. Laboratory testing of soil. Part 4: Determination of particle size distribution” was followed to perform the hydrometer test on quarry fine samples. Two samples were tested and the average was used for data analysis (Table S3).

**Figure 3:**
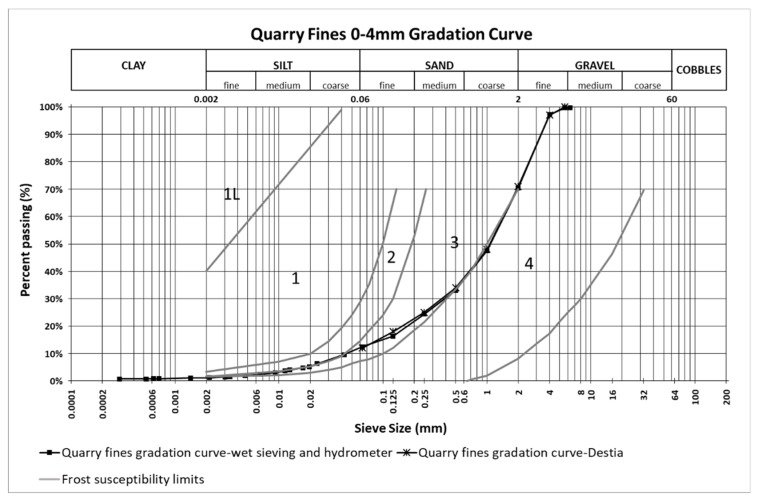
Estimation of frost susceptibility from the gradation curves of quarry fines according to ISSMGE [42]. Adapted with permission from [42]; 2005, Slunga, E., Saarelainen, S.

Figure 3 shows the gradation curve of quarry fines provided by the quarry plant, the gradation curve obtained from combining the results from wet sieving and the hydrometer tests, as well as the four regions defining different potentials of frost susceptibility for different granular materials. This chart is typically used to estimate the frost susceptibility of materials according to their gradation curves, based on the criterion proposed by the ISSMGE Technical Committee on Frost, which is also the guide for the Finnish Road Administration [42]. If the gradation curve stays within region 1 only, this material can be seen as frost susceptible, but in region 1L the susceptibility is low. Gradation curves falling completely in regions 2, 3, or 4 indicate non-frost-susceptibility. However, any curves spanning one region to another in the finer part (left), similar to the gradation curve of quarry fines shown in Figure 3, can be seen as frost susceptible. The highly consistent part of the gradation curves obtained from laboratory tests and provided by the quarry plant Destia, although only available for particles coarser than 0.063mm, have shown the reliability of the gradation information. The completed gradation curve a containing finer particle distribution (<0.063mm) indicates that frost susceptibility tests are greatly necessary for quarry fine materials since untreated frost susceptible material is not appropriate to be applied in base, subbase, or filter layers in pavements. Consequently, an improvement in the quarry fine materials to validate their application in pavement constructions, such as stabilization techniques, are highly recommended.

### 4.2. Density and Water Content

Granular material properties are closely related to moisture content. When compacted, the particles come together more closely and the dry density increases. The maximum dry density that can be achieved mainly depends on not only the effective compaction work but also the water content of the mixture. For a given degree of compaction work, there is typically an optimum water content at which the dry density reaches a maximum value for the non-free-draining materials. However, for a self-draining mixture, which is defined by the difference in water content before and after compaction being larger than 0.3%, may not have a well-defined water-density relationship. Therefore, a preliminary evaluation of the quarry fines to determine if they are a self-draining material is the first step to adoption.

**Figure 4:**
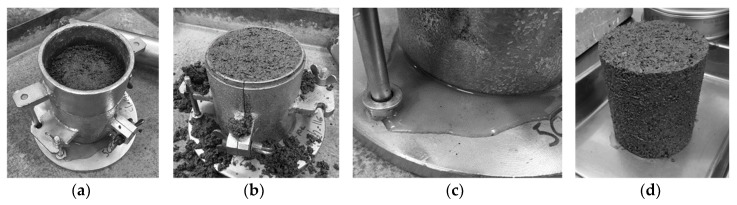
Laboratory reference density and water content by Proctor compaction method: (**a**) Specimen with mold during compactions; (**b**) Compacted quarry fine specimen in mold; (**c**) Self-draining water during the compaction; (**d**) Compacted quarry fine specimen for water content determination.

The European standard EN 13286-2: 2010 “Unbound and hydraulically bound mixtures. Part 2: Test methods for laboratory reference density and water content—Proctor compaction” [43] was followed to characterize the water-density relationship of quarry fines, as shown in Figure 4. According to the difference between initial and final water content before and after compaction, quarry fines can be categorized as a self-draining material with a maximum difference in water content of 1.6%. It is also interesting to note that such variations in water content due to compaction are also related to the initial water content of the quarry fines. If more water is added to achieve a higher initial water content, the mixture is more prone to draining the excess water during compaction and thus the water content difference is larger.

**Figure 5:**
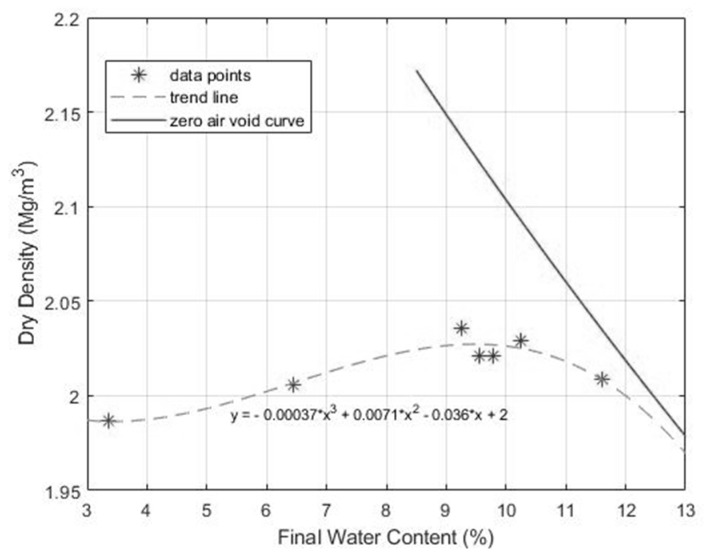
Variation of compacted dry density versus final water content.

Figure 5 and Table S4 present the relationship between the final water content and the dry density of the quarry fines. It is found that even though the quarry fines have self-draining properties, a maximum dry density still exists at the optimal water content of 9.3%. The maximum dry density of quarry fines that can be achieved from the Proctor test is 2.04 Mg/m^3^, as shown in Figure 5. However, in comparison to the zero air void curve, it can be seen that water content has limited influence on the dry density of quarry fines. All of these findings have shown combined characteristics of both self-draining and fine-graded granular materials and have also indicated the complicated properties of this material.

### 4.3. Permeability

Permeability is another dominant property of pavement base and subbase materials in pavement design since excessive moisture inside the pavement structure would bring adverse effects to the performance of the system. Water-related deteriorations in highway pavements have been commonly and widely observed in the past decades, and the inadequate drainage of base and subbase layers is one of the contributors. The permeability of quarry fine material, however, has not been well established in the past. The determination of the permeability of the quarry fine specimens is very necessary and could be used as a baseline to validate the application of this material as pavement base, subbase, or even other applications.

**Figure 6:**
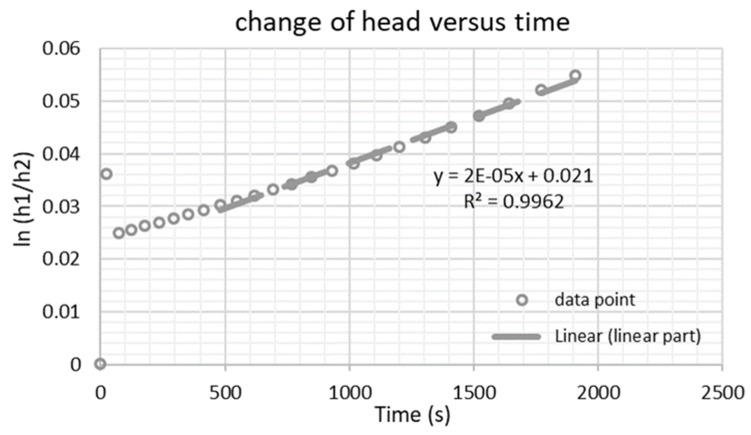
Change of head versus time during falling head permeability test.

Laboratory tests were conducted on unbound quarry fine specimens compacted at the optimum water content as determined by Proctor method. The falling head method provided by the Finnish Road and Water Engineering Board, which is suitable for fine-grained materials with intermediate-to-low permeability, was followed [44]. Whether the flow behavior of quarry fine specimens during the falling head test complies with Darcy’s Law based on laminar flow is the first key question that needed to be answered, considering that Darcy’s Law is adopted for permeability calculations in this method. To do so, a graph of change of head versus time was plotted to determine the linear part of the curve (if any) indicating the validity of Darcy’s law (Table S5 and Figure 6).

**Figure 7:**
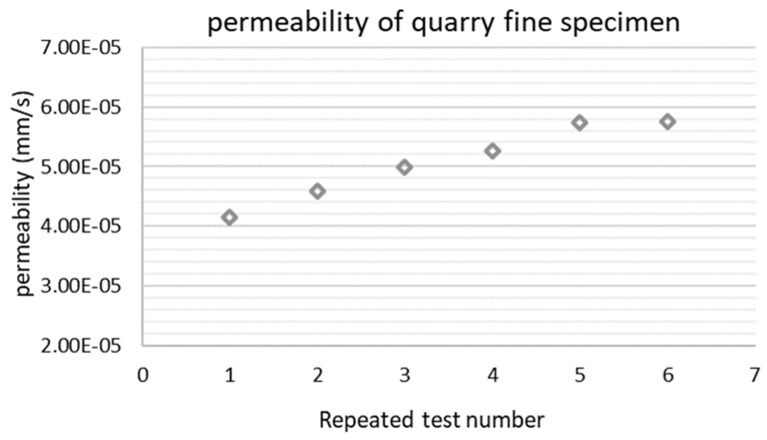
Permeability results of the unbound quarry fine specimen.

As indicated in Figure 6, not all the data points obtained from the falling head permeability test are appropriate to be used for calculating permeability based on Darcy’s law. The non-linear hydraulic gradient–velocity relationship at the beginning of the test shows that during this period, Darcy’s law is not applicable. Therefore, only data from the successive time period (around 500 s from the beginning till the end) was adopted for data analysis. Figure 7 shows the permeability results of the unbound quarry fine specimen from a series of successive repeated tests. The coefficient of permeability of the quarry fine specimen increases slightly and then levels off in the sixth repeated test, at 5.75 × 10^−5^ m/s. According to Federal Highway Administration (FHWA) guidelines, this coefficient of permeability for quarry fines with a 0–4 mm particle size is still far below the requirements for a drainage aggregate base, which should have a minimum permeability of 500 ft/day (1.75×10^−3^ m/s). Instead, it is comparable to the typical coefficient of permeability of fine gravel, coarse and medium sand, and dense-graded aggregate base (4.2 × 10^−5^ to 1.43 × 10^−4^ m/s) [45] and is slightly higher than many of the reported situations from experiments or in the field nowadays [46,47]. Generally, based on the laboratory-determined permeability properties of quarry fines and the literature data, it was found that the permeability of quarry fine specimens falls within the typical permeability ranges of commonly adopted conventional pavement base and subbase materials, thus it can be validated for associated applications from the view of permeability. Since there are hardly any permeability requirements for the filter layer, the validity of quarry fines as filter layer material from permeability aspect is not possible. However, considering the major function of filter layers is to prevent fines immigration and frost actions in cold regions, lower permeability is desirable and a higher degree of compaction is consequently recommended for this purpose.

### 4.4. Capillary Rise

In capillary theory, frost heave is closely related to the rate of temperature decrease, pore sizes between particles, and the hydraulic conductivity of the unfrozen part of the material [48]. If the material can easily draw water up to the frozen zone by capillary actions and also has a high hydraulic conductivity which facilitates the delivery of a large quantity of water, large ice lenses are able to form and it is possible that the material is frost-susceptible. Therefore, to better understand the frost susceptibility of quarry fine material, capillary rise test according to Finnish test method provided by the Road and Water Engineering Board was performed [44]. It was found that the capillary rise values for virgin quarry fine samples are in range of 0.70 to 0.85 m, with an average of 0.804 m, based on four repeated laboratory tests. This capillary rise value is just in accordance with the limitation set by the Finnish railway department regarding frost susceptibility, indicating that the virgin quarry fines is possibly frost-susceptible and improvements are necessary. The capillary rise test results are consistent with the gradation information and further verified the necessity for stabilization.

### 4.5. Frost Heave

According to the lab-determined gradation information as well as existing information on frost susceptibility (Figure 3), the quarry fines with a 0–4 mm particle size might be frost-susceptible. It was therefore necessary to investigate frost susceptibility with a frost heave test.

**Figure 8:**
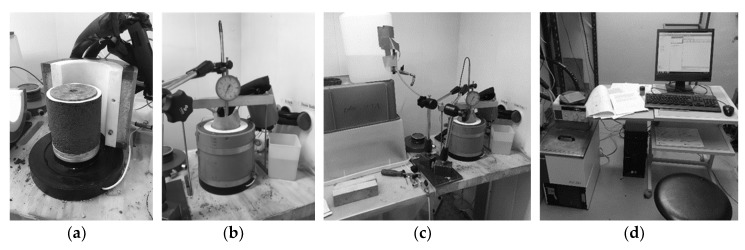
Frost heave test on quarry fine specimen: (**a**) Frozen quarry fine specimen mounted on the test apparatus; (**b**) Assembled specimen; (**c**) Assembled specimen with water supply; (**d**) Computer and temperature control system).

The frost heave test method description TPPT-R07 by VTT communities and infrastructure [49] was followed to test unbound quarry fines. Specimens of 100 mm diameter and 100 mm height were compacted at the optimal water content using the Proctor method to reach maximum dry density. The specimen, together with its mold, is then kept frozen overnight for conditioning. The extruded frozen specimen was then placed with filter paper and a stone on the bottom of the test cell (Figure 8), covered with a rubber membrane and the top cap. The rubber membrane was fixed to the cap and the bottom of the test cell with O-rings, similar to the procedures in a triaxial test. After applying a thin layer of silicone to the surface of the membrane, the insulated split barrels were greased to the specimen and membrane. External support rings were then tightened to ensure the supporting of insulated barrels. Finally, the displacement transducers and the loading frame were attached to the cap of the test cell.

A series of frost heave tests, including three individual frost heave tests and intermediate thawing processes of different surfacing loading conditions, were performed on the specimen. The conditions adopted in the frost heave and thawing processes are listed in Table 2. Since there are six temperature sensors embedded in the insulated barrels at different depths and two on each of the top cap and the bottom of the test cell, the temperature profile throughout the specimen could be recorded during the test, and the 0 °C isotherm (assumed as the freezing front) could be calculated by interpolation. Water intake from the bottom filter stone was allowed during the entire frost heave test to simulate the moisture transfer from ground water in practice.

**Figure 9:**
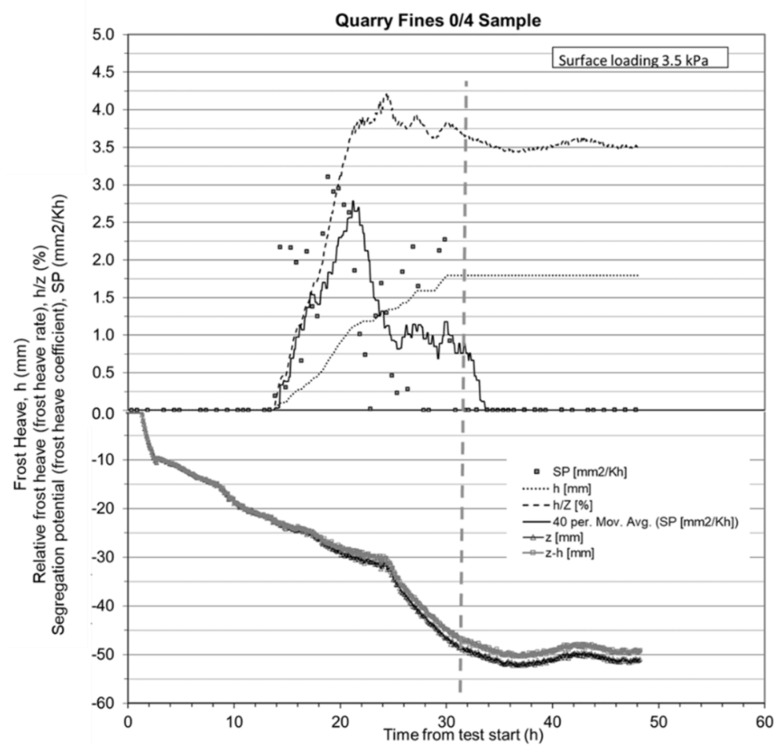
Frost heave parameters of a quarry fines (0–4mm) sample.

Figure 8 shows the quarry fine specimen and frost heave test apparatus as applied in this study. Figure 9 presents the total heave, depth of frost front during the test, and some frost susceptibility parameters and indicators of quarry fines as obtained from the test. All these data and results are derived from the first frost heave test with a surface loading of 3.5 kPa (Table S6).

There are several parameters and frost susceptibility indicators that can be derived from the frost heave test, as shown in Figure 9:

- *h* is the frost heave, or the change in height of the specimen during the test, in mm;
- *z* is the depth of frost penetration, which is the sum of the initial height and the frost heave minus the height of the unfrozen part of the specimen, in mm;
- *h*/*z* is the frost heave ratio, which is an indicator of the relative percentage of frost heaving from the depth of frost penetration, in %;
- *SP* is the segregation potential, or frost heave coefficient, which is the ratio between the frost heave rate and the actual temperature gradient over the frozen part of the specimen, in mm^2^/Kh.

As shown in Figure 9, the total frost heave (*h*) of the quarry fine specimen in the unloaded frost heave period can be divided into three stages. In the very first stage, from the beginning of the test to 13.67 h, there is no obvious frost heave observed, even though the temperature inside the specimen decreased significantly. In the second stage, when the temperature at the very top of specimen dropped to around zero, total heave started to increase strongly with time. During this stage, water inside the specimen froze and expanded against the particles, which resulted in a total height increase in the specimen. Finally, in the last stage when the temperature throughout the specimen reached equilibrium status, the total frost heave leveled off at around 1.79 mm. Accordingly, the depth of frost front (height of the unfrozen part) decreased with the origin at the very bottom of the specimen, or the depth of frost part of the specimen increased as the top plate of the specimen was kept cool by the circulation of cryomat-cooled liquid. The depth of frost penetration remained stable at about 50.0mm from the time when the temperature throughout the whole specimen was stable. The frost heave ratio is also plotted as *h*/*z* in Figure 9, showing that it increased and finally leveled off at 3.5% during the unloaded frost heave test.

The net depth of frost penetration (*z*–*h*), which is the depth of frost penetration minus frost heave, has the same changing tendency with time as for the depth of frost penetration. This curve is critical in finding out the segregation potential (*SP*) value for this quarry fine material and is used as a reference. The *SP*, however, was found to be a much more scattered data series than the other parameters, but the trend can still be approximated by moving average techniques. The *SP* value can be read from Figure 9 on the predictive moving average curve of the SP data, at the very first point where the net depth of frost penetration becomes stable. In this case, it was found that there was a transition period 30 h after the beginning of test, when the *SP* values suddenly decreased, becoming negligibly small. The *SP* value of quarry fine material in this study, was found to be just within this area. The exact point was obtained by looking for the point on the *z*–*h* curve that has the same *z*–*h* value as the average *z*–*h* value from this point and afterwards. This point was found to be at 32.09 h from the beginning of test, and the corresponding *SP* value at this point is 0.75 on the moving average curve. It should be noted that this SP value is conservative because afterwards the frost heave is stable and the SP value becomes negligible.

The segregation potential of the unbound quarry fines with 0–4 mm particle sizes is then determined to be 0.75. When compared to the frost susceptibility classification in the literature, as listed in Table 3, it appears that the segregation potential values indicats a frost class of low susceptibility. The results from the laboratory capillary test further verify this low potential for frost susceptibility, showing that the capillary rise of the quarry fines with a 0–4 mm particle size is around 70 to 85 mm (Officially the limit is 1.0 meter but nowadays the Finnish agencies are more prone to use stricter requirements). Even though the unbound quarry fine materials is not very frost susceptible, it is still to some extent susceptible to frost actions and thus not safe to be used for pavements as it is, especially in cold regions. In this regard, stabilization techniques are necessary to improve the frost susceptibility of quarry fines and enable its applications in pavement base, subbase, or filter layers.

## 5. Discussion and Conclusions

The aggregate source depletion, as well as the demand for a carbon-neutral society and circular economy, all promote the high-volume utilization of secondary materials such as quarry fines. In this research, a feasibility assessment including a series of laboratory tests and analyses were conducted to evaluate the properties of quarry fine materials to determine if this type of material could be qualified for pavement base, subbase, or filter layers, especially in high volume applications, as well as to evaluate the durability of quarry fines in term of frost susceptibility. The main conclusions are listed as follows:

- The wet sieving method is more appropriate for determining the gradation curve of quarry fines with a 0–4 mm grain size. Based on gradation information obtained from laboratory sieving and hydrometer tests, the quarry fines used in this study can be classified as well-graded with the gradation characteristics of both fine- and dense-graded material. It also shows that virgin quarry fines can satisfy the requirements for a filter layer but not for a base or subbase, and indicates that quarry fines might be frost susceptible.
- The virgin quarry fines showed self-draining properties. Nevertheless, a maximum dry density exists at the optimal water content of 9.3%, even though only a very limited influence of water content can be observed on the dry density.
- The coefficient of permeability of unstabilized quarry fine specimen was around 5.75×10-5 m/s as determined by the falling head method. This value falls within the typical permeability ranges of commonly adopted conventional pavement base and subbase materials and can be validated for application in base, subbase, and other layers from the view of permeability. To be validated for filter layers, a higher degree of compaction is desirable to prevent fines immigration and frost actions in cold regions.
- According to the frost heave test results, it was found that the changing of total frost heave is closely related to the temperature and depth of frost penetration throughout the specimen.
- The quarry fine specimen in the unloaded frost heave test can be classified into the low frost-susceptibility class according to the segregation potential values. In addition, gradation information and capillary rise test results have all verified this finding. As a result, it is concluded that the unstabilized quarry fines should be classified as frost-susceptible to ensure sound and reliable design and good performance in the long-run. To improve the frost-susceptible properties of quarry fines, stabilization techniques are necessary to qualify their application as pavement construction materials. Further research is undergoing and will be presented in the future.

## Figures and Tables

**Table 1:** The amount of quarry fines at Lemminkäinen Infra Oy [18].

Grain Size (mm)	2016 (tons)	2017 (tons)	Accumulation/Year (tons)	Accumulation since 2017 (%)
0/3	824,300	875,800	51,500	5.9
0/6	118,000	127,300	9,300	7.3
In total	942,300	1,003,100	60,800	6.1

**Table 2:** Frost heave and thawing conditions adopted in the test.

Step	Loading Condition	Temperature		Time Period	Water Level of External Water Reservoir
		Top	Bottom		
Preliminary freezing	Unloaded *	−3 °C	+1 °C	24 h	N/A
Thawing and preloading	20 kPa	+3 °C	+3 °C	24 h, or until constant height **	Top level of specimen
Frost heave	unloaded	−3 °C	+1 °C	until zero net frost penetration *** (at least 24 h)	At the middle of specimen height
Intermediate thawing	20 kPa	−0.5 °C	+15 °C	until constant height	At the middle of specimen height
1st frost heave	20 kPa	−3 °C	+1 °C	until zero net frost penetration (at least 24 h)	At the middle of specimen height
Intermediate thawing	40 kPa	−0.5 °C	+15 °C	until constant height	At the middle of specimen height
2nd frost heave	40 kPa	−3 °C	+1 °C	until zero net frost penetration (at least 24 h)	At the middle of specimen height

Notes: Unloaded * means the specimen is loaded by the frame only (3.5 kPa); Constant height ** means the height of the specimen is unchanged for more than four hours; Net frost penetration *** is the frost penetration minus frost heave, for more than four hours.

**Table 3:** Determination of the frost susceptibility of a soil type [42,50].

Frost Class	Segregation Potential SP (mm^2^/Kh)
Negligible	<0.5
Low	0.5–1.5
Medium	1.5–3.0
Strong	>3.0

## Supplementary Materials

The following are available online at https://www.mdpi.com/1996-1944/12/8/1209/s1, supplementary materials.docx. Table S1: Particle size distribution of quarry fines by sieving method, Table S2: Gradation limits for different layers, Table S3: Hydrometer test results of particle size distribution (0–0.063 mm), Table S4: Dry density versus final water content from Proctor test, Table S5: Permeability test results, Table S6: Frost heave test results.
