# Supplementary material for: Assessment for Sustainable Use of Quarry Fines as Pavement Construction Materials: Part I—Description of Basic Quarry Fine Properties
Source: Materials (Basel). 2019 Apr 12;12(8):1209. doi: 10.3390/ma12081209 (PMC6514762; doi:10.3390/ma12081209)
Supplement: Supplementary file 1 [file materials-12-01209-s001.pdf]

materials

Supplementary Materials: Assessment for Sustainable Use of Quarry Fines as Pavement Construction Materials: Part I—Description of Basic Quarry Fine Properties

Yinning Zhang *, Leena Katariina Korkiala–Tanttu, Henry Gustavsson and Amandine Miksic

Department of Civil Engineering, Aalto University, Helsinki 02150, Finland;
leena.korkiala–tanttu@aalto.fi (L.K.K.–T.); henry.gustavsson@aalto.fi (H.G.); amandine.miksic@aalto.fi (A.M.)
* Correspondence: yinning.zhang@aalto.fi

1. Particle Size Distribution by Sieving Method

Table S1. Particle size distribution of quarry fines by sieving method.

Sieving Size	Total Percent Passing (Wet)	Total Percent Passing (Dry)
0.06	0.12	0.09
0.13	0.19	0.16
0.25	0.26	0.24
0.50	0.34	0.33
1.00	0.49	0.48
2.00	0.71	0.70
4.00	0.97	0.97
5.60	1.00	1.00
6.30	1.00	1.00

Table S2. Gradation limits for different layers.

Sieving Size	Subbase Lower Limits	Subbase Upper Limits	Sieving Size	Finnish Base Lower Limits	European Base Lower Limits	Finnish Base Upper Limits	European Base Upper Limits	Sieving Size	Filter Layer Lower Limits	Filter Layer Upper Limits	Filter Layer c Limits
	Percent Passing	Percent Passing		Percent Passing	Percent Passing	Percent Passing	Percent Passing		Percent Passing	Percent Passing	Percent Passing
125.00	0.85	1.00	16.00	0.58	0.63	0.70	0.77	0.02	0.00	0.05	0.03
31.50	0.46	1.00	8.00	0.39	0.43	0.51	0.57	0.06	0.00	0.15	0.07
16.00	0.32	1.00	4.00	0.26	0.30	0.38	0.42	0.13	0.00	0.32	0.12
4.00	0.12	0.85	2.00	0.17	0.22	0.28	0.33	0.25	0.00	0.70	0.20
1.00	0.03	0.52	1.00	0.11	0.15	0.21	0.30	0.50	0.00	1.00	0.32
0.06	0.00	0.09	0.50	0.05	0.05	0.15	0.15	1.00	0.20	1.00	0.50
0.02	0.00	0.03	N/A	N/A	N/A	N/A	N/A	2.00	0.50	1.00	0.70
N/A	N/A	N/A	N/A	N/A	N/A	N/A	N/A	8.00	0.85	1.00	N/A
N/A	N/A	N/A	N/A	N/A	N/A	N/A	N/A	31.50	1.00	1.00	N/A

1.1. Hydrometer Test

Table S3. Hydrometer test results of particle size distribution (0–0.063 mm).

Sample 1									
Time	Reading	Temperature(°C)	η	H	H_r	t	d_i	R_d	K
1 min	1.02	23.10	0.93	43.24	103.43	1.00	0.04	24.00	77.00
4 min	1.02	23.10	0.93	65.76	125.95	4.00	0.02	15.50	49.73
6 min	1.01	23.10	0.93	72.39	132.58	6.00	0.02	13.00	41.71
8 min	1.01	23.10	0.93	75.04	135.23	8.00	0.02	12.00	38.50
15 min	1.01	23.00	0.94	80.34	140.53	15.00	0.01	10.00	32.08
19 min	1.01	22.90	0.94	82.99	143.18	19.00	0.01	9.00	28.88
30 min	1.01	22.90	0.94	87.76	147.95	30.00	0.01	7.20	23.10

1 h	1.01	22.90	0.94	91.21	151.39	60.00	0.01	5.90	18.93
2 h	1.00	22.90	0.94	94.12	154.31	120.00	0.00	4.80	15.40
4 h	1.00	22.90	0.94	96.24	156.43	240.00	0.00	4.00	12.83
5 h	1.00	22.90	0.94	97.56	157.75	300.00	0.00	3.50	11.23
10 h	1.00	22.50	0.95	98.89	159.08	600.00	0.00	3.00	9.63
24 h	1.00	22.00	0.96	100.22	160.40	1440.00	0.00	2.50	8.02
4 day	1.00	22.40	0.95	101.28	161.46	5760.00	0.00	2.10	6.74
5 day	1.00	23.10	0.93	101.54	161.73	7200.00	0.00	2.00	6.42
7 day	1.00	23.10	0.93	101.81	161.99	10080.00	0.00	1.90	6.10
23 day	1.00	22.30	0.95	101.81	161.99	33120.00	0.00	1.90	6.10
**Sample 2**									
**Time**	**Reading**	**Temperature(°C)**	**η**	***H***	***H*r**	***t***	***d*i**	***R*d**	***K***
1 min	1.02	23.00	0.94	43.24	103.43	1.00	0.042	24.00	77.00
4 min	1.02	23.00	0.94	64.44	124.63	4.00	0.023	16.00	51.34
6 min	1.01	23.00	0.94	72.39	132.58	6.00	0.020	13.00	41.71
8 min	1.01	23.00	0.94	75.04	135.23	8.00	0.017	12.00	38.50
15 min	1.01	23.00	0.94	80.34	140.53	15.00	0.013	10.00	32.08
19 min	1.01	23.00	0.94	82.46	142.65	19.00	0.011	9.20	29.52
30 min	1.01	23.00	0.94	85.91	146.09	30.00	0.009	7.90	25.35
1 h	1.01	23.00	0.94	90.68	150.86	60.00	0.007	6.10	19.57
2 h	1.01	22.90	0.94	93.59	153.78	120.00	0.005	5.00	16.04
4 h	1.00	22.90	0.94	95.98	156.16	240.00	0.003	4.10	13.15
5 h	1.00	22.90	0.94	96.77	156.96	300.00	0.003	3.80	12.19
10 h	1.00	22.50	0.95	98.62	158.81	600.00	0.002	3.10	9.95
24 h	1.00	22.00	0.96	99.42	159.61	1440.00	0.001	2.80	8.98
4 day	1.00	22.40	0.95	101.01	161.20	5760.00	0.001	2.20	7.06
5 day	1.00	23.10	0.93	101.54	161.73	7200.00	0.001	2.00	6.42
7 day	1.00	23.10	0.93	101.81	161.99	10080.00	0.001	1.90	6.10
23 day	1.00	22.40	0.95	101.81	161.99	33120.00	0.000	1.90	6.10

1.2. Proctor Test

Table S4. Dry density versus final water content from Proctor test.

Sample	Final Water Content (%)	Dry Density (mg/m³)
#1	9.566	2.021
#2	9.775	2.021
#3	3.368	1.986
#4	10.253	2.029
#5	11.600	2.009
#6	9.258	2.036
#7	6.439	2.006

1.3. Permeability Test

Table S5. Permeability test results.

Time (s)	Accumulated Time (s)	ln(*h*1/*h*2)
24.22	24.22	0.036149
49.67	73.89	0.024846

52.85	126.74	0.025479
54.02	180.76	0.026145
57.18	237.94	0.026847
58.06	296.00	0.027588
57.82	353.82	0.028371
64.74	418.56	0.029199
64.19	482.75	0.030077
67.28	550.03	0.03101
71.94	621.97	0.032003
72.03	694.00	0.033061
77.41	771.41	0.034191
79.27	850.68	0.035402
83.14	933.82	0.036701
85.22	1019.04	0.0381
90.71	1109.75	0.039609
94.83	1204.58	0.041243
101.77	1306.35	0.043017
104.71	1411.06	0.044951
113.25	1524.31	0.047068
119.43	1643.74	0.049393
128.03	1771.77	0.05196
136.60	1908.37	−0.054808

1.4. Frost Heave Test

Table S6. Frost heave test results.

Time (h)	Transitional (mm)	Cover (°C)	Sensor 1 (°C)	Sensor 2 (°C)	Sensor 3 (°C)	Sensor 4 (°C)	Sensor 5 (°C)	Sensor 6 (°C)	Base (°C)
0.000	0.00000	3.586	3.318	3.403	3.399	3.352	3.342	3.453	3.165
0.083	0.00000	3.421	3.299	3.378	3.404	3.354	3.380	3.460	3.119
0.167	−0.00007	3.096	3.281	3.392	3.389	3.354	3.360	3.310	1.639
0.250	−0.00010	2.640	3.190	3.374	3.344	3.298	3.203	2.964	1.462
0.333	−0.00017	2.333	3.061	3.277	3.299	3.220	3.038	2.764	1.424
0.417	−0.00017	2.099	2.931	3.179	3.181	3.081	2.918	2.658	1.403
0.500	−0.00016	1.818	2.839	3.059	3.083	2.991	2.817	2.568	1.385
0.583	−0.00014	1.508	2.727	2.944	2.977	2.882	2.699	2.467	1.377
0.667	−0.00004	1.242	2.604	2.861	2.900	2.803	2.638	2.425	1.348
0.750	−0.00012	0.992	2.482	2.727	2.795	2.731	2.548	2.347	1.354
0.833	−0.00014	0.786	2.346	2.625	2.679	2.636	2.492	2.323	1.332
0.917	−0.00013	0.625	2.231	2.530	2.590	2.536	2.415	2.275	1.311
1.000	−0.00005	0.526	2.138	2.415	2.500	2.487	2.390	2.231	1.339
1.083	−0.00015	0.332	1.976	2.315	2.397	2.394	2.309	2.180	1.325
1.167	−0.00017	0.180	1.866	2.210	2.324	2.321	2.240	2.141	1.288
1.250	−0.00018	0.063	1.779	2.103	2.226	2.251	2.205	2.118	1.304
1.333	−0.00027	−0.096	1.658	2.023	2.143	2.207	2.156	2.091	1.306
1.417	−0.00028	−0.229	1.555	1.925	2.058	2.123	2.111	2.035	1.289
1.500	−0.00024	−0.330	1.468	1.831	1.983	2.060	2.053	2.014	1.296
1.583	−0.00025	−0.429	1.364	1.755	1.919	2.017	2.014	1.988	1.295
1.667	−0.00022	−0.527	1.273	1.665	1.840	1.943	1.979	1.966	1.271
1.750	−0.00025	−0.618	1.167	1.578	1.780	1.897	1.929	1.938	1.281
1.834	−0.00020	−0.682	1.110	1.529	1.697	1.820	1.880	1.908	1.297
1.917	−0.00018	−0.760	1.036	1.441	1.631	1.783	1.835	1.900	1.290
2.000	−0.00019	−0.834	0.960	1.361	1.576	1.734	1.787	1.873	1.251
2.084	−0.00020	−0.892	0.896	1.301	1.518	1.708	1.761	1.844	1.239
2.167	−0.00017	−0.929	0.856	1.254	1.487	1.644	1.744	1.843	1.274

2.250	−0.00024	−1.011	0.784	1.211	1.420	1.610	1.686	1.803	1.263
2.334	−0.00025	−1.058	0.705	1.150	1.352	1.559	1.658	1.787	1.215
2.417	−0.00015	−1.115	0.642	1.061	1.305	1.515	1.629	1.759	1.244
2.500	−0.00013	−1.128	0.613	1.049	1.274	1.496	1.616	1.743	1.248
2.584	−0.00027	−1.205	0.573	0.987	1.204	1.432	1.563	1.714	1.244
2.667	−0.00015	−1.236	0.528	0.954	1.190	1.404	1.575	1.703	1.244
2.750	−0.00011	−1.167	0.604	0.932	1.168	1.394	1.551	1.706	1.259
2.834	−0.00026	−1.206	0.663	0.969	1.172	1.378	1.544	1.710	1.251
2.917	−0.00018	−1.259	0.670	0.991	1.164	1.361	1.520	1.677	1.247
3.000	−0.00014	−1.267	0.661	0.989	1.149	1.350	1.503	1.670	1.250
3.084	−0.00012	−1.259	0.667	1.004	1.175	1.362	1.504	1.677	1.254
3.167	−0.00023	−1.282	0.643	1.004	1.164	1.351	1.509	1.668	1.239
3.250	−0.00025	−1.278	0.644	0.999	1.130	1.373	1.483	1.682	1.210
3.334	−0.00024	−1.280	0.620	0.984	1.138	1.340	1.476	1.665	1.266
3.417	−0.00012	−1.279	0.597	0.952	1.150	1.350	1.486	1.663	1.268
3.500	−0.00021	−1.276	0.594	0.959	1.148	1.340	1.495	1.646	1.221
3.584	−0.00018	−1.273	0.555	0.931	1.119	1.304	1.479	1.642	1.251
3.667	−0.00020	−1.253	0.552	0.935	1.109	1.298	1.459	1.652	1.261
3.750	−0.00011	−1.230	0.545	0.908	1.089	1.321	1.466	1.657	1.242
3.834	−0.00027	−1.238	0.530	0.921	1.110	1.317	1.455	1.646	1.234
3.917	−0.00018	−1.248	0.488	0.882	1.076	1.297	1.460	1.662	1.257
4.000	−0.00019	−1.229	0.476	0.876	1.069	1.295	1.465	1.636	1.241
4.084	−0.00015	−1.203	0.457	0.875	1.074	1.266	1.449	1.630	1.245
4.167	−0.00022	−1.195	0.484	0.863	1.068	1.290	1.454	1.651	1.269
4.250	−0.00026	−1.210	0.448	0.848	1.044	1.257	1.446	1.629	1.249
4.334	−0.00027	−1.203	0.450	0.825	1.040	1.267	1.416	1.650	1.253
4.417	−0.00025	−1.202	0.444	0.825	1.032	1.252	1.436	1.628	1.262
4.500	−0.00027	−1.178	0.408	0.812	1.044	1.243	1.439	1.633	1.254
4.584	−0.00018	−1.201	0.418	0.809	1.015	1.235	1.406	1.614	1.223
4.667	−0.00024	−1.226	0.390	0.793	0.998	1.235	1.402	1.613	1.236
4.750	−0.00022	−1.201	0.399	0.771	1.019	1.234	1.401	1.603	1.231
4.834	−0.00016	−1.218	0.374	0.772	0.994	1.221	1.393	1.597	1.232
4.917	−0.00030	−1.243	0.354	0.782	0.991	1.216	1.378	1.617	1.226
5.001	−0.00021	−1.249	0.349	0.765	0.981	1.199	1.376	1.598	1.236
5.084	−0.00013	−1.244	0.346	0.769	0.968	1.207	1.395	1.603	1.209
5.167	−0.00017	−1.240	0.322	0.755	0.975	1.225	1.396	1.591	1.240
5.251	−0.00025	−1.283	0.331	0.743	0.968	1.196	1.401	1.595	1.202
5.334	−0.00023	−1.271	0.302	0.716	0.940	1.197	1.392	1.624	1.235
5.417	−0.00022	−1.277	0.291	0.751	0.952	1.182	1.360	1.591	1.242
5.501	−0.00017	−1.295	0.277	0.729	0.961	1.174	1.355	1.584	1.243
5.584	−0.00006	−1.289	0.273	0.724	0.931	1.170	1.361	1.581	1.231
5.667	−0.00016	−1.329	0.271	0.714	0.949	1.183	1.361	1.593	1.247
5.751	−0.00024	−1.324	0.252	0.689	0.911	1.172	1.358	1.585	1.227
5.834	−0.00026	−1.305	0.222	0.703	0.923	1.160	1.358	1.593	1.236
5.917	−0.00024	−1.324	0.219	0.665	0.925	1.159	1.346	1.571	1.230
6.001	−0.00017	−1.330	0.220	0.667	0.910	1.149	1.335	1.567	1.231
6.084	−0.00023	−1.340	0.220	0.660	0.888	1.147	1.354	1.579	1.216
6.167	−0.00023	−1.350	0.203	0.658	0.897	1.127	1.354	1.584	1.213
6.251	−0.00025	−1.324	0.182	0.653	0.922	1.150	1.338	1.575	1.237
6.334	−0.00035	−1.352	0.171	0.637	0.883	1.165	1.363	1.583	1.224
6.417	−0.00020	−1.359	0.155	0.648	0.900	1.128	1.346	1.571	1.219
6.501	−0.00021	−1.373	0.132	0.627	0.864	1.140	1.330	1.566	1.192
6.584	−0.00017	−1.339	0.145	0.612	0.861	1.132	1.332	1.566	1.254
6.667	−0.00028	−1.360	0.140	0.648	0.874	1.111	1.353	1.565	1.216
6.751	−0.00021	−1.376	0.119	0.611	0.848	1.144	1.337	1.570	1.248
6.834	−0.00027	−1.383	0.128	0.616	0.847	1.125	1.310	1.572	1.223
6.917	−0.00024	−1.371	0.118	0.621	0.860	1.118	1.331	1.593	1.217
7.001	−0.00013	−1.337	0.089	0.621	0.859	1.109	1.333	1.580	1.246
7.084	−0.00018	−1.383	0.097	0.606	0.844	1.098	1.324	1.562	1.193
7.167	−0.00027	−1.377	0.099	0.581	0.846	1.099	1.316	1.579	1.237
7.251	−0.00026	−1.359	0.078	0.611	0.825	1.095	1.322	1.559	1.242
7.334	−0.00019	−1.323	0.098	0.605	0.817	1.099	1.324	1.573	1.230
7.417	−0.00032	−1.363	0.074	0.583	0.839	1.105	1.325	1.564	1.234

7.501	–0.00020	–1.352	0.051	0.588	0.816	1.075	1.318	1.569	1.232
7.584	–0.00016	–1.354	0.052	0.566	0.828	1.093	1.306	1.568	1.244
7.667	–0.00018	–1.350	0.061	0.579	0.827	1.098	1.322	1.580	1.240
7.751	–0.00032	–1.361	0.024	0.561	0.810	1.092	1.300	1.569	1.231
7.834	–0.00023	–1.343	0.041	0.555	0.817	1.080	1.295	1.550	1.212
7.917	–0.00017	–1.377	0.016	0.587	0.804	1.084	1.298	1.546	1.216
8.001	–0.00014	–1.364	0.029	0.569	0.813	1.087	1.310	1.554	1.231
8.084	–0.00015	–1.359	0.017	0.565	0.824	1.080	1.304	1.552	1.227
8.167	–0.00010	–1.387	–0.002	0.517	0.815	1.086	1.302	1.554	1.249
8.251	–0.00019	–1.400	–0.003	0.537	0.800	1.084	1.288	1.556	1.236
8.334	–0.00017	–1.400	–0.011	0.526	0.802	1.064	1.294	1.547	1.217
8.418	–0.00011	–1.404	0.010	0.549	0.787	1.084	1.314	1.559	1.228
8.501	–0.00021	–1.457	–0.013	0.531	0.779	1.066	1.299	1.550	1.220
8.584	–0.00014	–1.484	–0.013	0.544	0.781	1.048	1.284	1.545	1.196
8.668	–0.00019	–1.497	–0.016	0.531	0.776	1.064	1.280	1.542	1.218
8.751	–0.00009	–1.495	–0.030	0.517	0.790	1.071	1.286	1.549	1.217
8.834	–0.00021	–1.543	–0.043	0.515	0.773	1.054	1.288	1.519	1.232
8.918	–0.00015	–1.547	–0.025	0.494	0.774	1.052	1.267	1.540	1.220
9.001	–0.00015	–1.570	–0.046	0.502	0.767	1.072	1.273	1.528	1.220
9.084	–0.00013	–1.551	–0.065	0.507	0.759	1.053	1.285	1.551	1.204
9.168	–0.00019	–1.597	–0.065	0.522	0.786	1.048	1.267	1.557	1.232
9.251	–0.00014	–1.592	–0.083	0.490	0.782	1.044	1.270	1.535	1.224
9.334	–0.00016	–1.606	–0.086	0.492	0.759	1.020	1.285	1.532	1.193
9.418	–0.00008	–1.606	–0.075	0.501	0.765	1.058	1.287	1.549	1.214
9.501	–0.00020	–1.616	–0.122	0.473	0.768	1.042	1.288	1.543	1.221
9.584	–0.00010	–1.637	–0.116	0.484	0.754	1.035	1.259	1.534	1.224
9.668	–0.00004	–1.653	–0.108	0.493	0.736	1.036	1.259	1.543	1.224
9.751	–0.00010	–1.663	–0.126	0.485	0.762	1.044	1.268	1.543	1.253
9.834	–0.00017	–1.678	–0.125	0.472	0.738	1.040	1.279	1.544	1.231
9.918	–0.00016	–1.697	–0.122	0.493	0.736	1.040	1.290	1.509	1.223
10.001	–0.00017	–1.699	–0.168	0.462	0.741	1.037	1.262	1.520	1.226
10.084	–0.00024	–1.712	–0.153	0.462	0.754	1.024	1.267	1.527	1.215
10.168	–0.00022	–1.713	–0.158	0.461	0.740	1.032	1.269	1.525	1.217
10.251	–0.00025	–1.749	–0.150	0.433	0.740	1.017	1.249	1.537	1.228
10.334	–0.00003	–1.740	–0.163	0.440	0.727	1.033	1.255	1.531	1.222
10.418	0.00000	–1.730	–0.173	0.444	0.707	1.022	1.261	1.530	1.226
10.501	–0.00010	–1.720	–0.195	0.424	0.734	1.033	1.244	1.548	1.201
10.584	–0.00003	–1.760	–0.185	0.432	0.711	1.018	1.260	1.516	1.212
10.668	–0.00007	–1.753	–0.181	0.443	0.727	1.001	1.241	1.527	1.199
10.751	–0.00002	–1.772	–0.187	0.423	0.724	1.011	1.274	1.532	1.229
10.834	–0.00002	–1.727	–0.185	0.440	0.723	1.021	1.283	1.556	1.254
10.918	–0.00013	–1.781	–0.200	0.415	0.703	1.021	1.280	1.527	1.236
11.001	–0.00001	–1.793	–0.215	0.403	0.711	1.018	1.250	1.550	1.197
11.084	0.00001	–1.789	–0.210	0.402	0.708	1.008	1.259	1.539	1.215
11.168	0.00000	–1.758	–0.214	0.417	0.707	1.001	1.255	1.513	1.237
11.251	–0.00003	–1.742	–0.218	0.404	0.711	1.016	1.256	1.543	1.231
11.334	–0.00001	–1.764	–0.235	0.391	0.701	1.006	1.259	1.521	1.212
11.418	–0.00009	–1.772	–0.227	0.404	0.701	1.009	1.241	1.520	1.220
11.501	–0.00009	–1.766	–0.227	0.390	0.703	1.009	1.258	1.535	1.226
11.584	–0.00016	–1.722	–0.200	0.368	0.701	1.011	1.265	1.539	1.209
11.668	–0.00013	–1.768	–0.213	0.365	0.688	0.995	1.238	1.521	1.173
11.751	–0.00008	–1.764	–0.245	0.368	0.716	1.000	1.233	1.538	1.239
11.835	–0.00015	–1.744	–0.240	0.386	0.699	1.005	1.240	1.518	1.202
11.918	–0.00007	–1.749	–0.232	0.366	0.672	0.999	1.269	1.526	1.234
12.001	–0.00017	–1.758	–0.241	0.388	0.687	0.985	1.258	1.531	1.211
12.085	–0.00011	–1.752	–0.234	0.345	0.685	0.991	1.239	1.550	1.206
12.168	–0.00010	–1.759	–0.242	0.370	0.663	0.980	1.239	1.529	1.216
12.251	–0.00014	–1.747	–0.241	0.348	0.670	0.992	1.242	1.523	1.207
12.335	–0.00025	–1.732	–0.246	0.355	0.676	0.994	1.253	1.525	1.179
12.418	–0.00012	–1.760	–0.264	0.360	0.672	0.997	1.228	1.510	1.219
12.501	–0.00018	–1.753	–0.255	0.329	0.653	0.993	1.238	1.513	1.206
12.585	–0.00021	–1.763	–0.260	0.343	0.662	0.993	1.236	1.539	1.225
12.668	–0.00009	–1.734	–0.254	0.333	0.667	0.999	1.222	1.518	1.220

12.751	−0.00018	−1.740	−0.249	0.351	0.665	0.978	1.243	1.533	1.210
12.835	−0.00010	−1.750	−0.256	0.334	0.674	0.984	1.248	1.512	1.190
12.918	−0.00002	−1.763	−0.267	0.334	0.648	0.984	1.217	1.538	1.198
13.001	−0.00002	−1.764	−0.257	0.318	0.662	0.972	1.240	1.518	1.230
13.085	−0.00007	−1.736	−0.272	0.308	0.645	0.989	1.218	1.537	1.228
13.168	−0.00009	−1.750	−0.265	0.345	0.647	0.971	1.238	1.523	1.214
13.251	0.00009	−1.771	−0.262	0.313	0.664	0.961	1.243	1.535	1.224
13.335	0.00011	−1.763	−0.266	0.309	0.653	0.992	1.234	1.501	1.230
13.418	0.00006	−1.760	−0.272	0.319	0.643	0.993	1.232	1.516	1.216
13.501	0.00004	−1.777	−0.259	0.317	0.666	0.966	1.229	1.527	1.187
13.585	0.00019	−1.811	−0.281	0.303	0.643	0.976	1.214	1.532	1.209
13.668	0.00019	−1.828	−0.299	0.278	0.626	0.962	1.208	1.498	1.187
13.751	0.00024	−1.810	−0.299	0.288	0.643	0.979	1.227	1.509	1.212
13.835	0.00803	−1.807	−0.281	0.301	0.641	0.972	1.239	1.520	1.221
13.918	0.02896	−1.847	−0.303	0.293	0.633	0.965	1.232	1.501	1.200
14.001	0.04444	−1.839	−0.308	0.288	0.636	0.959	1.213	1.505	1.205
14.085	0.05718	−1.853	−0.312	0.269	0.641	0.941	1.212	1.515	1.211
14.168	0.07286	−1.838	−0.309	0.278	0.619	0.981	1.221	1.524	1.214
14.251	0.08678	−1.849	−0.309	0.282	0.630	0.966	1.204	1.527	1.224
14.335	0.09400	−1.857	−0.334	0.265	0.634	0.972	1.216	1.519	1.183
14.418	0.09939	−1.862	−0.323	0.261	0.624	0.965	1.211	1.514	1.195
14.501	0.10536	−1.843	−0.344	0.272	0.625	0.955	1.220	1.529	1.207
14.585	0.10543	−1.832	−0.327	0.291	0.631	0.965	1.226	1.525	1.208
14.668	0.10567	−1.838	−0.309	0.258	0.626	0.963	1.217	1.500	1.218
14.751	0.10565	−1.849	−0.358	0.251	0.612	0.933	1.202	1.508	1.191
14.835	0.10570	−1.835	−0.346	0.227	0.596	0.946	1.202	1.512	1.189
14.918	0.11146	−1.819	−0.328	0.261	0.617	0.940	1.219	1.509	1.223
15.002	0.12982	−1.824	−0.337	0.260	0.608	0.947	1.215	1.508	1.202
15.085	0.14596	−1.822	−0.348	0.239	0.611	0.947	1.216	1.511	1.200
15.168	0.16147	−1.811	−0.341	0.259	0.594	0.971	1.205	1.505	1.203
15.252	0.17484	−1.774	−0.339	0.239	0.600	0.936	1.200	1.508	1.219
15.335	0.18592	−1.773	−0.331	0.235	0.602	0.939	1.201	1.493	1.206
15.418	0.19463	−1.789	−0.341	0.243	0.607	0.946	1.191	1.506	1.187
15.502	0.21195	−1.785	−0.345	0.227	0.602	0.934	1.187	1.493	1.217
15.585	0.22571	−1.761	−0.348	0.233	0.586	0.943	1.202	1.522	1.220
15.668	0.23768	−1.752	−0.340	0.227	0.613	0.946	1.222	1.511	1.219
15.752	0.24549	−1.783	−0.349	0.218	0.622	0.949	1.211	1.506	1.204
15.835	0.25634	−1.762	−0.337	0.203	0.578	0.924	1.185	1.492	1.219
15.918	0.26670	−1.748	−0.344	0.225	0.612	0.952	1.199	1.506	1.228
16.002	0.27159	−1.710	−0.331	0.245	0.594	0.927	1.195	1.496	1.224
16.085	0.27290	−1.764	−0.325	0.222	0.578	0.915	1.208	1.518	1.240
16.168	0.27317	−1.760	−0.337	0.225	0.579	0.948	1.199	1.486	1.203
16.252	0.27382	−1.776	−0.340	0.225	0.596	0.929	1.184	1.491	1.227
16.335	0.28011	−1.757	−0.340	0.210	0.597	0.925	1.184	1.524	1.229
16.418	0.29725	−1.767	−0.355	0.231	0.589	0.926	1.191	1.500	1.207
16.502	0.30922	−1.805	−0.354	0.205	0.563	0.901	1.194	1.495	1.186
16.585	0.32005	−1.775	−0.344	0.220	0.571	0.895	1.176	1.496	1.231
16.668	0.33172	−1.771	−0.354	0.207	0.582	0.925	1.182	1.498	1.204
16.752	0.34121	−1.758	−0.357	0.217	0.559	0.907	1.189	1.500	1.224
16.835	0.35624	−1.821	−0.351	0.178	0.568	0.913	1.193	1.503	1.228
16.918	0.35894	−1.815	−0.349	0.192	0.563	0.904	1.184	1.498	1.200
17.002	0.35909	−1.800	−0.371	0.203	0.587	0.907	1.185	1.486	1.203
17.085	0.36899	−1.805	−0.363	0.208	0.565	0.915	1.193	1.503	1.229
17.168	0.38026	−1.827	−0.367	0.201	0.564	0.925	1.174	1.470	1.207
17.252	0.39204	−1.825	−0.366	0.196	0.567	0.926	1.187	1.488	1.196
17.335	0.40611	−1.808	−0.369	0.202	0.571	0.916	1.194	1.505	1.212
17.418	0.41957	−1.799	−0.376	0.177	0.570	0.918	1.201	1.508	1.214
17.502	0.42953	−1.866	−0.368	0.187	0.580	0.913	1.172	1.504	1.192
17.585	0.43143	−1.894	−0.380	0.176	0.564	0.908	1.200	1.499	1.197
17.668	0.43616	−1.897	−0.394	0.166	0.555	0.893	1.173	1.485	1.205
17.752	0.44227	−1.903	−0.391	0.170	0.542	0.893	1.180	1.494	1.211
17.835	0.45299	−1.947	−0.387	0.160	0.556	0.899	1.160	1.515	1.227
17.918	0.46608	−1.973	−0.419	0.156	0.568	0.908	1.173	1.489	1.187

18.002	0.47881	−1.975	−0.436	0.162	0.547	0.914	1.178	1.493	1.234
18.085	0.48858	−2.002	−0.422	0.164	0.548	0.909	1.200	1.505	1.209
18.168	0.50413	−2.000	−0.426	0.161	0.535	0.892	1.177	1.503	1.222
18.252	0.52127	−2.016	−0.456	0.141	0.554	0.890	1.159	1.484	1.182
18.335	0.53888	−2.000	−0.462	0.123	0.526	0.862	1.166	1.485	1.210
18.419	0.55574	−2.018	−0.437	0.129	0.550	0.879	1.169	1.484	1.203
18.502	0.57506	−2.031	−0.456	0.157	0.535	0.893	1.172	1.480	1.185
18.585	0.59649	−2.036	−0.441	0.118	0.522	0.885	1.174	1.467	1.213
18.669	0.61430	−2.025	−0.460	0.111	0.535	0.877	1.159	1.475	1.213
18.752	0.63240	−2.032	−0.489	0.135	0.523	0.898	1.185	1.482	1.235
18.835	0.65272	−2.044	−0.486	0.110	0.521	0.898	1.162	1.477	1.198
18.919	0.67468	−2.079	−0.474	0.117	0.530	0.870	1.143	1.488	1.212
19.002	0.69171	−2.062	−0.482	0.101	0.520	0.872	1.158	1.480	1.200
19.085	0.70677	−2.042	−0.492	0.116	0.535	0.900	1.158	1.505	1.190
19.169	0.72444	−2.048	−0.467	0.127	0.533	0.878	1.171	1.492	1.197
19.252	0.74331	−2.088	−0.507	0.095	0.513	0.868	1.149	1.479	1.209
19.335	0.75978	−2.087	−0.516	0.097	0.504	0.875	1.140	1.471	1.218
19.419	0.77689	−2.052	−0.493	0.097	0.511	0.841	1.169	1.467	1.209
19.502	0.79725	−2.056	−0.516	0.096	0.538	0.867	1.156	1.493	1.194
19.585	0.81834	−2.101	−0.515	0.087	0.511	0.877	1.142	1.478	1.209
19.669	0.83696	−2.101	−0.518	0.086	0.502	0.857	1.162	1.482	1.210
19.752	0.85215	−2.092	−0.518	0.077	0.524	0.844	1.159	1.460	1.213
19.835	0.86749	−2.066	−0.497	0.100	0.526	0.855	1.153	1.476	1.204
19.919	0.88922	−2.089	−0.522	0.086	0.500	0.849	1.123	1.480	1.195
20.002	0.90639	−2.107	−0.523	0.089	0.509	0.828	1.146	1.478	1.218
20.085	0.92073	−2.099	−0.524	0.054	0.504	0.865	1.137	1.470	1.205
20.169	0.93433	−2.063	−0.539	0.079	0.488	0.861	1.163	1.474	1.219
20.252	0.95039	−2.101	−0.534	0.080	0.493	0.859	1.144	1.465	1.197
20.335	0.96425	−2.081	−0.521	0.061	0.468	0.848	1.149	1.477	1.186
20.419	0.97810	−2.085	−0.529	0.048	0.503	0.858	1.133	1.483	1.202
20.502	0.99445	−2.064	−0.516	0.065	0.491	0.872	1.158	1.481	1.218
20.585	1.00975	−2.094	−0.534	0.066	0.495	0.851	1.151	1.472	1.183
20.669	1.02398	−2.096	−0.546	0.054	0.481	0.843	1.146	1.465	1.227
20.752	1.03858	−2.067	−0.547	0.061	0.477	0.838	1.141	1.477	1.218
20.835	1.05716	−2.078	−0.538	0.065	0.476	0.852	1.162	1.451	1.237
20.919	1.07255	−2.086	−0.535	0.058	0.462	0.848	1.126	1.489	1.213
21.002	1.08829	−2.107	−0.531	0.041	0.478	0.833	1.131	1.447	1.214
21.085	1.10261	−2.093	−0.556	0.038	0.491	0.839	1.136	1.459	1.198
21.169	1.11453	−2.086	−0.539	0.048	0.481	0.860	1.141	1.457	1.210
21.252	1.11626	−2.111	−0.555	0.040	0.490	0.842	1.147	1.463	1.196
**21.335**	1.12278	−2.094	−0.536	0.057	0.480	0.830	1.146	1.469	1.204
21.419	1.12878	−2.091	−0.559	0.021	0.474	0.841	1.122	1.476	1.202
21.502	1.13971	−2.092	−0.542	0.044	0.463	0.844	1.140	1.476	1.229
21.585	1.14090	−2.095	−0.557	0.055	0.462	0.826	1.128	1.488	1.188
21.669	1.14340	−2.100	−0.574	0.030	0.461	0.837	1.124	1.469	1.216
21.752	1.14704	−2.073	−0.566	0.024	0.465	0.861	1.130	1.475	1.196
21.836	1.15858	−2.108	−0.551	0.046	0.470	0.843	1.149	1.467	1.201
**21.919**	1.16530	−2.106	−0.575	0.038	0.449	0.819	1.143	1.464	1.184
22.002	1.16646	−2.119	−0.561	0.011	0.461	0.826	1.119	1.473	1.202
22.086	1.17590	−2.114	−0.578	0.045	0.445	0.839	1.114	1.482	1.241
22.169	1.18350	−2.133	−0.590	0.014	0.438	0.843	1.130	1.475	1.204
22.252	1.18391	−2.121	−0.589	−0.002	0.436	0.828	1.123	1.463	1.214
22.336	1.18415	−2.127	−0.566	0.020	0.440	0.821	1.133	1.477	1.183
22.419	1.18432	−2.159	−0.596	0.024	0.457	0.836	1.134	1.463	1.200
22.502	1.18428	−2.168	−0.563	0.014	0.437	0.836	1.150	1.465	1.194
22.586	1.18424	−2.149	−0.531	0.027	0.471	0.857	1.145	1.483	1.206
22.669	1.18455	−2.150	−0.553	0.029	0.470	0.872	1.159	1.494	1.248
22.752	1.18460	−2.190	−0.589	0.005	0.455	0.842	1.128	1.470	1.202
22.836	1.18474	−2.145	−0.589	0.010	0.421	0.828	1.128	1.463	1.203
22.919	1.18475	−2.138	−0.580	0.009	0.464	0.819	1.137	1.462	1.220
23.002	1.18604	−2.142	−0.564	0.012	0.441	0.811	1.135	1.456	1.212
23.086	1.19069	−2.129	−0.595	0.006	0.429	0.819	1.124	1.458	1.210
23.169	1.19911	−2.100	−0.574	0.011	0.437	0.816	1.123	1.447	1.203

23.252	1.21563	−2.093	−0.564	−0.009	0.428	0.823	1.119	1.464	1.202
23.336	1.22737	−2.101	−0.568	0.007	0.424	0.812	1.117	1.446	1.220
23.419	1.23704	−2.078	−0.559	−0.005	0.439	0.818	1.100	1.469	1.212
23.502	1.25213	−2.080	−0.571	−0.006	0.437	0.812	1.106	1.464	1.212
23.586	1.26372	−2.073	−0.593	−0.009	0.404	0.794	1.113	1.439	1.219
23.669	1.27253	−2.043	−0.583	0.012	0.410	0.796	1.098	1.455	1.212
23.752	1.27730	−2.017	−0.543	0.009	0.426	0.799	1.095	1.437	1.201
23.836	1.28053	−2.038	−0.572	−0.034	0.414	0.796	1.086	1.440	1.188
23.919	1.28064	−2.036	−0.567	−0.015	0.405	0.772	1.089	1.436	1.219
24.002	1.28074	−2.032	−0.556	−0.020	0.402	0.789	1.110	1.447	1.196
24.086	1.29393	−2.067	−0.563	0.006	0.420	0.781	1.100	1.438	1.185
24.169	1.30454	−2.097	−0.557	−0.004	0.402	0.809	1.113	1.470	1.200
24.252	1.31626	−2.180	−0.589	−0.006	0.414	0.786	1.104	1.466	1.184
24.336	1.32652	−2.229	−0.590	−0.005	0.406	0.782	1.108	1.450	1.172
24.419	1.34202	−2.302	−0.591	−0.038	0.424	0.811	1.091	1.440	1.197
24.502	1.34391	−2.358	−0.625	−0.023	0.415	0.794	1.093	1.428	1.184
24.586	1.34385	−2.404	−0.642	−0.032	0.412	0.773	1.095	1.441	1.201
24.669	1.34410	−2.482	−0.669	−0.035	0.420	0.786	1.092	1.460	1.227
24.752	1.34434	−2.529	−0.677	−0.048	0.394	0.773	1.086	1.436	1.191
24.836	1.34438	−2.578	−0.717	−0.070	0.381	0.760	1.096	1.454	1.215
24.919	1.34435	−2.636	−0.741	−0.064	0.383	0.779	1.093	1.433	1.197
25.003	1.34447	−2.672	−0.758	−0.109	0.389	0.768	1.077	1.443	1.199
25.086	1.34812	−2.707	−0.772	−0.096	0.383	0.761	1.079	1.463	1.215
25.169	1.35313	−2.730	−0.771	−0.111	0.382	0.787	1.093	1.440	1.226
25.253	1.35347	−2.810	−0.787	−0.147	0.371	0.768	1.071	1.441	1.196
25.336	1.35358	−2.829	−0.851	−0.138	0.346	0.758	1.084	1.454	1.197
25.419	1.35386	−2.816	−0.834	−0.137	0.355	0.793	1.077	1.455	1.198
25.503	1.37290	−2.866	−0.857	−0.144	0.352	0.774	1.103	1.436	1.166
25.586	1.38614	−2.893	−0.870	−0.167	0.339	0.739	1.058	1.427	1.204
25.669	1.39844	−2.897	−0.888	−0.196	0.327	0.759	1.067	1.416	1.198
25.753	1.41277	−2.914	−0.902	−0.189	0.317	0.768	1.087	1.419	1.191
25.836	1.42669	−2.961	−0.902	−0.213	0.324	0.737	1.054	1.442	1.192
25.919	1.42870	−2.961	−0.944	−0.214	0.334	0.759	1.070	1.417	1.216
26.003	1.43053	−2.967	−0.940	−0.219	0.317	0.760	1.066	1.420	1.208
26.086	1.43075	−3.002	−0.935	−0.226	0.317	0.747	1.048	1.432	1.192
26.169	1.43101	−3.021	−0.965	−0.247	0.301	0.745	1.047	1.424	1.201
26.253	1.43107	−3.063	−0.971	−0.237	0.295	0.738	1.059	1.426	1.182
26.336	1.43813	−3.084	−0.973	−0.261	0.300	0.718	1.067	1.415	1.181
26.419	1.45474	−3.095	−0.997	−0.272	0.303	0.744	1.065	1.423	1.224
26.503	1.47196	−3.122	−1.008	−0.289	0.295	0.726	1.065	1.425	1.182
26.586	1.48582	−3.137	−1.021	−0.296	0.271	0.721	1.037	1.421	1.203
26.669	1.49868	−3.136	−1.030	−0.313	0.284	0.722	1.044	1.417	1.208
26.753	1.51213	−3.154	−1.031	−0.289	0.251	0.739	1.042	1.411	1.211
26.836	1.52473	−3.183	−1.037	−0.293	0.226	0.720	1.043	1.428	1.176
26.919	1.54017	−3.226	−1.073	−0.329	0.241	0.715	1.043	1.430	1.183
27.003	1.55700	−3.245	−1.075	−0.317	0.257	0.695	1.049	1.427	1.191
27.086	1.57421	−3.247	−1.064	−0.306	0.216	0.707	1.059	1.425	1.184
27.169	1.58501	−3.298	−1.108	−0.336	0.241	0.698	1.037	1.423	1.209
27.253	1.59151	−3.310	−1.101	−0.349	0.197	0.695	1.036	1.415	1.207
27.336	1.59165	−3.311	−1.101	−0.357	0.220	0.704	1.044	1.424	1.173
27.419	1.59154	−3.317	−1.126	−0.369	0.217	0.698	1.047	1.396	1.173
27.503	1.59175	−3.362	−1.129	−0.376	0.203	0.704	1.010	1.401	1.165
27.586	1.59188	−3.365	−1.142	−0.379	0.183	0.700	1.022	1.402	1.203
27.669	1.59180	−3.361	−1.179	−0.390	0.167	0.671	1.020	1.394	1.192
27.753	1.59165	−3.372	−1.173	−0.408	0.191	0.683	1.035	1.402	1.186
27.836	1.59184	−3.391	−1.164	−0.391	0.171	0.693	1.018	1.391	1.169
27.919	1.59189	−3.397	−1.170	−0.415	0.186	0.672	1.012	1.388	1.180
28.003	1.59191	−3.393	−1.168	−0.431	0.177	0.676	1.024	1.407	1.211
28.086	1.59177	−3.370	−1.189	−0.413	0.152	0.679	1.004	1.413	1.180
28.169	1.59173	−3.416	−1.206	−0.454	0.144	0.680	1.000	1.388	1.171
28.253	1.59190	−3.403	−1.190	−0.455	0.129	0.690	0.984	1.397	1.195
28.336	1.59185	−3.426	−1.234	−0.434	0.119	0.662	0.991	1.380	1.183
28.420	1.59180	−3.386	−1.218	−0.442	0.110	0.660	1.004	1.391	1.199

28.503	1.59174	−3.426	−1.210	−0.468	0.114	0.648	0.992	1.374	1.191
28.586	1.59188	−3.439	−1.228	−0.463	0.113	0.641	0.986	1.389	1.169
28.670	1.59189	−3.453	−1.238	−0.472	0.101	0.646	0.980	1.391	1.188
28.753	1.59184	−3.438	−1.236	−0.481	0.112	0.648	0.985	1.390	1.166
28.836	1.59184	−3.456	−1.249	−0.491	0.098	0.652	1.002	1.382	1.193
28.920	1.59822	−3.489	−1.244	−0.495	0.088	0.646	0.968	1.398	1.179
29.003	1.61178	−3.486	−1.261	−0.486	0.069	0.630	0.983	1.403	1.170
29.086	1.62278	−3.491	−1.259	−0.483	0.080	0.627	0.981	1.372	1.178
29.170	1.63985	−3.474	−1.270	−0.504	0.066	0.633	0.984	1.412	1.190
29.253	1.65857	−3.486	−1.300	−0.508	0.041	0.609	0.971	1.372	1.177
29.336	1.67430	−3.501	−1.283	−0.523	0.061	0.613	0.958	1.386	1.173
29.420	1.69019	−3.489	−1.286	−0.518	0.047	0.612	0.955	1.367	1.178
29.503	1.70402	−3.474	−1.297	−0.533	0.037	0.640	0.960	1.390	1.184
29.586	1.72320	−3.504	−1.304	−0.522	0.031	0.591	0.960	1.362	1.183
29.670	1.73601	−3.509	−1.308	−0.556	0.034	0.596	0.961	1.364	1.155
29.753	1.74726	−3.528	−1.290	−0.540	0.019	0.593	0.943	1.368	1.167
29.836	1.75864	−3.462	−1.295	−0.547	0.005	0.574	0.952	1.372	1.195
29.920	1.77624	−3.492	−1.311	−0.542	0.011	0.577	0.945	1.373	1.180
30.003	1.78934	−3.510	−1.307	−0.542	0.005	0.566	0.950	1.381	1.174
30.086	1.79299	−3.518	−1.299	−0.575	0.007	0.569	0.943	1.358	1.162
30.170	1.79286	−3.511	−1.316	−0.565	0.002	0.558	0.951	1.366	1.172
30.253	1.79262	−3.462	−1.319	−0.574	−0.011	0.565	0.926	1.383	1.166
30.336	1.79292	−3.492	−1.314	−0.576	−0.018	0.559	0.946	1.353	1.162
30.420	1.79296	−3.506	−1.314	−0.585	−0.040	0.550	0.945	1.377	1.160
30.503	1.79287	−3.487	−1.335	−0.580	−0.019	0.538	0.924	1.362	1.158
30.586	1.79277	−3.493	−1.314	−0.588	−0.014	0.557	0.934	1.370	1.179
30.670	1.79299	−3.513	−1.327	−0.579	−0.048	0.554	0.953	1.370	1.161
30.753	1.79305	−3.487	−1.334	−0.568	−0.050	0.540	0.936	1.365	1.178
30.836	1.79306	−3.474	−1.322	−0.592	−0.041	0.540	0.913	1.362	1.179
30.920	1.79294	−3.446	−1.320	−0.579	−0.035	0.528	0.941	1.375	1.189
31.003	1.79291	−3.469	−1.308	−0.598	−0.049	0.528	0.920	1.361	1.136
31.086	1.79313	−3.472	−1.348	−0.593	−0.054	0.516	0.938	1.365	1.181
31.170	1.79306	−3.467	−1.327	−0.603	−0.074	0.535	0.923	1.366	1.167
31.253	1.79300	−3.441	−1.331	−0.598	−0.072	0.531	0.925	1.363	1.188
31.336	1.79288	−3.441	−1.310	−0.609	−0.061	0.520	0.927	1.352	1.181
31.420	1.79307	−3.477	−1.340	−0.601	−0.071	0.514	0.904	1.347	1.180
31.503	1.79302	−3.471	−1.326	−0.634	−0.084	0.498	0.904	1.354	1.165
31.586	1.79307	−3.432	−1.346	−0.621	−0.069	0.505	0.901	1.346	1.171
31.670	1.79281	−3.410	−1.350	−0.590	−0.088	0.521	0.908	1.358	1.163
31.753	1.79304	−3.458	−1.341	−0.618	−0.102	0.515	0.919	1.360	1.158
31.837	1.79304	−3.437	−1.338	−0.623	−0.081	0.489	0.894	1.330	1.156
31.920	1.79308	−3.446	−1.350	−0.610	−0.082	0.491	0.910	1.327	1.155
32.003	1.79297	−3.398	−1.331	−0.619	−0.075	0.490	0.918	1.337	1.164
32.087	1.79320	−3.436	−1.302	−0.631	−0.087	0.489	0.895	1.350	1.164
32.170	1.79319	−3.438	−1.349	−0.607	−0.116	0.491	0.895	1.337	1.117
32.253	1.79309	−3.425	−1.357	−0.604	−0.085	0.460	0.898	1.333	1.151
32.337	1.79302	−3.377	−1.332	−0.618	−0.089	0.489	0.916	1.336	1.173
32.420	1.79316	−3.432	−1.322	−0.612	−0.105	0.492	0.876	1.312	1.178
32.503	1.79319	−3.424	−1.332	−0.610	−0.125	0.464	0.871	1.342	1.184
32.587	1.79313	−3.435	−1.333	−0.656	−0.111	0.480	0.883	1.329	1.160
32.670	1.79313	−3.431	−1.330	−0.624	−0.109	0.463	0.897	1.348	1.165
32.753	1.79303	−3.437	−1.331	−0.634	−0.105	0.473	0.872	1.333	1.154
32.837	1.79333	−3.440	−1.329	−0.628	−0.126	0.477	0.883	1.322	1.150
32.920	1.79333	−3.443	−1.341	−0.642	−0.128	0.471	0.874	1.337	1.165
33.003	1.79321	−3.422	−1.335	−0.637	−0.103	0.483	0.873	1.341	1.182
33.087	1.79298	−3.419	−1.344	−0.618	−0.124	0.476	0.880	1.321	1.171
33.170	1.79329	−3.456	−1.347	−0.632	−0.133	0.448	0.875	1.334	1.160
33.253	1.79331	−3.453	−1.350	−0.639	−0.126	0.444	0.862	1.352	1.165
33.337	1.79319	−3.427	−1.359	−0.640	−0.141	0.475	0.881	1.315	1.164
33.420	1.79302	−3.436	−1.357	−0.641	−0.142	0.445	0.880	1.326	1.148
33.503	1.79339	−3.464	−1.344	−0.644	−0.157	0.459	0.868	1.328	1.164
33.587	1.79331	−3.456	−1.353	−0.655	−0.139	0.464	0.876	1.326	1.116
33.670	1.79332	−3.453	−1.341	−0.641	−0.129	0.456	0.863	1.323	1.179

33.753	1.79312	–3.433	–1.374	–0.643	–0.149	0.457	0.876	1.345	1.190
33.837	1.79343	–3.470	–1.349	–0.688	–0.159	0.436	0.865	1.323	1.122
33.920	1.79333	–3.470	–1.370	–0.664	–0.149	0.422	0.858	1.324	1.137
34.003	1.79324	–3.481	–1.356	–0.652	–0.130	0.424	0.877	1.325	1.146
34.087	1.79315	–3.475	–1.374	–0.679	–0.152	0.438	0.854	1.315	1.158
34.170	1.79339	–3.486	–1.379	–0.665	–0.173	0.423	0.855	1.320	1.161
34.253	1.79349	–3.490	–1.385	–0.684	–0.166	0.440	0.872	1.322	1.168
34.337	1.79348	–3.472	–1.380	–0.661	–0.163	0.413	0.869	1.328	1.165
34.420	1.79323	–3.469	–1.375	–0.649	–0.166	0.437	0.862	1.325	1.171
34.503	1.79345	–3.490	–1.382	–0.697	–0.180	0.420	0.841	1.311	1.163
34.587	1.79352	–3.514	–1.358	–0.683	–0.164	0.406	0.842	1.327	1.155
34.670	1.79351	–3.499	–1.395	–0.685	–0.176	0.418	0.847	1.311	1.137
34.753	1.79337	–3.485	–1.386	–0.678	–0.144	0.415	0.858	1.306	1.164
34.837	1.79358	–3.492	–1.398	–0.694	–0.172	0.414	0.857	1.313	1.160
34.920	1.79363	–3.490	–1.395	–0.695	–0.172	0.409	0.831	1.301	1.164
35.004	1.79359	–3.486	–1.391	–0.685	–0.182	0.394	0.826	1.331	1.172
35.087	1.79337	–3.450	–1.375	–0.677	–0.171	0.404	0.841	1.305	1.163
35.170	1.79353	–3.474	–1.384	–0.718	–0.194	0.401	0.843	1.296	1.171
35.254	1.79366	–3.460	–1.401	–0.699	–0.196	0.381	0.844	1.295	1.161
35.337	1.79359	–3.450	–1.398	–0.698	–0.198	0.419	0.830	1.305	1.172
35.420	1.79337	–3.432	–1.369	–0.682	–0.199	0.384	0.838	1.316	1.185
35.504	1.79360	–3.435	–1.385	–0.688	–0.202	0.381	0.811	1.312	1.157
35.587	1.79368	–3.436	–1.380	–0.689	–0.193	0.375	0.840	1.303	1.151
35.670	1.79364	–3.426	–1.376	–0.693	–0.188	0.394	0.841	1.327	1.189
35.754	1.79341	–3.398	–1.375	–0.678	–0.179	0.390	0.834	1.308	1.182
35.837	1.79363	–3.415	–1.383	–0.709	–0.174	0.398	0.829	1.284	1.167
35.920	1.79366	–3.419	–1.372	–0.691	–0.189	0.362	0.815	1.294	1.141
36.004	1.79373	–3.393	–1.368	–0.691	–0.184	0.378	0.844	1.320	1.129
36.087	1.79353	–3.356	–1.382	–0.669	–0.203	0.394	0.838	1.313	1.129
36.170	1.79365	–3.384	–1.329	–0.684	–0.178	0.389	0.823	1.285	1.155
36.254	1.79376	–3.405	–1.353	–0.699	–0.217	0.382	0.828	1.298	1.153
36.337	1.79379	–3.369	–1.372	–0.685	–0.211	0.371	0.824	1.298	1.144
36.420	1.79377	–3.357	–1.323	–0.694	–0.190	0.381	0.811	1.312	1.167
36.504	1.79365	–3.366	–1.349	–0.666	–0.191	0.374	0.815	1.318	1.168
36.587	1.79397	–3.347	–1.352	–0.677	–0.197	0.370	0.818	1.311	1.158
36.670	1.79400	–3.345	–1.349	–0.666	–0.177	0.339	0.822	1.277	1.158
36.754	1.79392	–3.330	–1.344	–0.701	–0.206	0.368	0.841	1.319	1.152
36.837	1.79371	–3.323	–1.322	–0.678	–0.204	0.368	0.818	1.303	1.159
36.920	1.79394	–3.322	–1.340	–0.681	–0.189	0.364	0.795	1.289	1.158
37.004	1.79390	–3.300	–1.335	–0.676	–0.197	0.349	0.808	1.291	1.166
37.087	1.79388	–3.276	–1.314	–0.662	–0.180	0.348	0.811	1.298	1.188
37.170	1.79366	–3.293	–1.306	–0.660	–0.187	0.376	0.812	1.286	1.148
37.254	1.79394	–3.312	–1.327	–0.660	–0.174	0.364	0.801	1.298	1.172
37.337	1.79396	–3.270	–1.328	–0.671	–0.179	0.352	0.810	1.301	1.142
37.420	1.79387	–3.261	–1.315	–0.643	–0.211	0.376	0.822	1.289	1.138
37.504	1.79371	–3.247	–1.301	–0.659	–0.188	0.360	0.818	1.303	1.169
37.587	1.79392	–3.264	–1.320	–0.671	–0.204	0.376	0.804	1.311	1.148
37.670	1.79406	–3.273	–1.330	–0.659	–0.191	0.356	0.819	1.277	1.144
**37.754**	1.79398	–3.242	–1.298	–0.662	–0.167	0.374	0.806	1.307	1.141
37.837	1.79366	–3.202	–1.296	–0.659	–0.186	0.358	0.836	1.311	1.193
37.920	1.79396	–3.249	–1.283	–0.659	–0.182	0.354	0.817	1.297	1.157
38.004	1.79400	–3.230	–1.289	–0.661	–0.174	0.355	0.833	1.303	1.163
38.087	1.79389	–3.204	–1.289	–0.640	–0.186	0.357	0.811	1.292	1.169
38.170	1.79385	–3.170	–1.281	–0.630	–0.169	0.377	0.833	1.284	1.186
38.254	1.79405	–3.188	–1.270	–0.639	–0.167	0.350	0.823	1.283	1.166
38.337	1.79392	–3.198	–1.267	–0.637	–0.183	0.338	0.803	1.291	1.149
38.421	1.79390	–3.139	–1.257	–0.642	–0.182	0.370	0.804	1.292	1.188
38.504	1.79389	–3.164	–1.248	–0.615	–0.172	0.354	0.827	1.307	1.189
38.587	1.79403	–3.154	–1.231	–0.608	–0.159	0.387	0.815	1.311	1.178
38.671	1.79406	–3.151	–1.263	–0.612	–0.167	0.343	0.827	1.304	1.159
38.754	1.79404	–3.102	–1.244	–0.594	–0.173	0.365	0.815	1.289	1.165
38.837	1.79394	–3.111	–1.239	–0.608	–0.169	0.362	0.836	1.282	1.176
38.921	1.79408	–3.131	–1.236	–0.584	–0.182	0.362	0.808	1.294	1.184

39.004	1.79407	−3.124	−1.245	−0.605	−0.169	0.367	0.813	1.305	1.175
39.087	1.79403	−3.108	−1.249	−0.607	−0.158	0.368	0.820	1.287	1.185
39.171	1.79371	−3.050	−1.227	−0.597	−0.144	0.375	0.840	1.302	1.160
39.254	1.79403	−3.120	−1.214	−0.617	−0.151	0.357	0.818	1.289	1.160
39.337	1.79397	−3.080	−1.224	−0.598	−0.155	0.348	0.824	1.303	1.170
39.421	1.79401	−3.069	−1.216	−0.578	−0.168	0.349	0.839	1.312	1.167
39.504	1.79394	−3.034	−1.210	−0.584	−0.150	0.367	0.842	1.300	1.188
39.587	1.79392	−3.072	−1.190	−0.587	−0.142	0.360	0.820	1.314	1.164
39.671	1.79407	−3.058	−1.197	−0.572	−0.145	0.365	0.811	1.314	1.160
39.754	1.79409	−3.065	−1.195	−0.592	−0.148	0.357	0.826	1.286	1.168
39.837	1.79396	−3.009	−1.182	−0.575	−0.140	0.371	0.815	1.300	1.190
39.921	1.79390	−3.039	−1.200	−0.580	−0.143	0.370	0.837	1.327	1.169
40.004	1.79404	−3.059	−1.182	−0.562	−0.157	0.369	0.821	1.317	1.149
40.087	1.79407	−3.039	−1.184	−0.600	−0.141	0.383	0.836	1.312	1.172
40.171	1.79397	−3.027	−1.191	−0.569	−0.141	0.350	0.813	1.314	1.156
40.254	1.79391	−2.972	−1.164	−0.552	−0.131	0.366	0.839	1.309	1.151
40.337	1.79388	−3.009	−1.162	−0.555	−0.143	0.383	0.830	1.322	1.194
40.421	1.79401	−2.996	−1.173	−0.586	−0.133	0.373	0.841	1.316	1.183
40.504	1.79398	−2.992	−1.150	−0.566	−0.141	0.382	0.839	1.320	1.172
40.587	1.79396	−2.949	−1.151	−0.545	−0.119	0.375	0.809	1.329	1.156
40.671	1.79383	−2.962	−1.152	−0.543	−0.120	0.389	0.826	1.322	1.176
40.754	1.79398	−2.983	−1.129	−0.544	−0.120	0.369	0.820	1.308	1.194
40.837	1.79405	−2.975	−1.147	−0.561	−0.128	0.364	0.809	1.296	1.174
40.921	1.79397	−2.944	−1.130	−0.539	−0.116	0.390	0.830	1.325	1.181
41.004	1.79382	−2.906	−1.138	−0.538	−0.113	0.392	0.823	1.311	1.170
41.087	1.79404	−2.958	−1.149	−0.527	−0.100	0.399	0.837	1.319	1.163
41.171	1.79404	−2.928	−1.128	−0.524	−0.130	0.379	0.831	1.309	1.168
41.254	1.79406	−2.929	−1.134	−0.523	−0.116	0.388	0.831	1.320	1.185
41.337	1.79396	−2.890	−1.101	−0.519	−0.097	0.394	0.835	1.338	1.147
41.421	1.79405	−2.938	−1.115	−0.534	−0.100	0.382	0.812	1.321	1.199
41.504	1.79397	−2.945	−1.111	−0.524	−0.134	0.375	0.835	1.314	1.162
41.587	1.79398	−2.912	−1.132	−0.533	−0.113	0.386	0.829	1.316	1.203
41.671	1.79384	−2.904	−1.104	−0.509	−0.122	0.400	0.834	1.298	1.175
41.754	1.79381	−2.913	−1.118	−0.513	−0.108	0.379	0.825	1.299	1.185
41.838	1.79406	−2.942	−1.122	−0.521	−0.103	0.383	0.839	1.306	1.170
41.921	1.79408	−2.934	−1.109	−0.531	−0.094	0.370	0.825	1.309	1.188
42.004	1.79388	−2.901	−1.119	−0.533	−0.100	0.402	0.826	1.304	1.171
42.088	1.79390	−2.940	−1.110	−0.525	−0.095	0.393	0.846	1.334	1.181
42.171	1.79400	−2.948	−1.105	−0.511	−0.105	0.397	0.836	1.286	1.187
42.254	1.79395	−2.930	−1.120	−0.504	−0.104	0.381	0.837	1.302	1.165
42.338	1.79402	−2.898	−1.110	−0.535	−0.090	0.387	0.823	1.308	1.184
42.421	1.79386	−2.892	−1.109	−0.525	−0.115	0.393	0.845	1.310	1.198
42.504	1.79406	−2.962	−1.111	−0.518	−0.094	0.382	0.820	1.306	1.167
42.588	1.79401	−2.953	−1.137	−0.515	−0.109	0.388	0.836	1.293	1.148
42.671	1.79397	−2.932	−1.117	−0.506	−0.110	0.380	0.833	1.308	1.191
42.754	1.79392	−2.906	−1.112	−0.518	−0.090	0.388	0.826	1.304	1.185
42.838	1.79394	−2.949	−1.090	−0.517	−0.116	0.384	0.822	1.298	1.186
42.921	1.79404	−2.950	−1.121	−0.515	−0.088	0.380	0.813	1.297	1.143
43.004	1.79404	−2.959	−1.137	−0.516	−0.093	0.393	0.852	1.312	1.184
43.088	1.79406	−2.967	−1.105	−0.525	−0.119	0.383	0.827	1.292	1.179
43.171	1.79383	−2.947	−1.143	−0.514	−0.101	0.418	0.834	1.303	1.195
43.254	1.79400	−2.996	−1.122	−0.536	−0.106	0.385	0.833	1.298	1.191
43.338	1.79400	−2.988	−1.130	−0.528	−0.102	0.378	0.836	1.296	1.174
43.421	1.79400	−3.000	−1.139	−0.528	−0.102	0.382	0.838	1.298	1.161
43.504	1.79395	−2.971	−1.165	−0.546	−0.103	0.403	0.829	1.300	1.181
43.588	1.79391	−2.991	−1.138	−0.536	−0.114	0.390	0.844	1.304	1.193
43.671	1.79407	−3.025	−1.158	−0.540	−0.091	0.386	0.826	1.289	1.168
43.754	1.79400	−3.038	−1.147	−0.530	−0.112	0.377	0.837	1.303	1.176
43.838	1.79403	−3.017	−1.156	−0.545	−0.122	0.375	0.845	1.295	1.175
43.921	1.79394	−3.002	−1.161	−0.537	−0.114	0.395	0.831	1.286	1.186
44.004	1.79398	−3.010	−1.150	−0.552	−0.102	0.382	0.846	1.299	1.183
44.088	1.79416	−3.037	−1.161	−0.546	−0.122	0.380	0.811	1.301	1.166
44.171	1.79405	−3.060	−1.165	−0.549	−0.112	0.375	0.810	1.309	1.175

44.254	1.79406	−3.047	−1.155	−0.538	−0.124	0.400	0.816	1.287	1.164
44.338	1.79394	−3.045	−1.166	−0.558	−0.112	0.378	0.828	1.309	1.151
44.421	1.79409	−3.079	−1.175	−0.560	−0.129	0.388	0.830	1.291	1.152
44.504	1.79409	−3.095	−1.167	−0.556	−0.123	0.374	0.817	1.293	1.174
44.588	1.79403	−3.081	−1.181	−0.557	−0.121	0.394	0.846	1.290	1.170
44.671	1.79391	−3.069	−1.203	−0.558	−0.134	0.383	0.826	1.304	1.186
44.754	1.79403	−3.121	−1.182	−0.564	−0.114	0.379	0.831	1.291	1.164
44.838	1.79399	−3.126	−1.177	−0.564	−0.147	0.385	0.809	1.284	1.146
44.921	1.79392	−3.106	−1.197	−0.566	−0.125	0.375	0.812	1.290	1.173
45.005	1.79379	−3.104	−1.204	−0.566	−0.138	0.390	0.827	1.299	1.175
45.088	1.79399	−3.152	−1.216	−0.572	−0.138	0.384	0.818	1.293	1.166
45.171	1.79385	−3.138	−1.218	−0.589	−0.156	0.373	0.819	1.287	1.158
45.255	1.79396	−3.118	−1.221	−0.588	−0.140	0.388	0.809	1.286	1.193
45.338	1.79373	−3.113	−1.207	−0.572	−0.143	0.398	0.828	1.305	1.188
45.421	1.79397	−3.164	−1.207	−0.576	−0.140	0.365	0.804	1.273	1.158
45.505	1.79400	−3.143	−1.235	−0.580	−0.151	0.371	0.821	1.286	1.169
45.588	1.79395	−3.142	−1.226	−0.606	−0.163	0.376	0.816	1.271	1.165
45.671	1.79374	−3.157	−1.221	−0.590	−0.136	0.378	0.831	1.292	1.171
45.755	1.79401	−3.168	−1.226	−0.589	−0.155	0.372	0.815	1.302	1.180
45.838	1.79400	−3.171	−1.239	−0.601	−0.156	0.367	0.806	1.270	1.150
45.921	1.79391	−3.130	−1.227	−0.583	−0.162	0.367	0.818	1.273	1.175
46.005	1.79389	−3.169	−1.220	−0.588	−0.170	0.381	0.830	1.288	1.146
46.088	1.79404	−3.137	−1.245	−0.606	−0.154	0.381	0.798	1.289	1.151
46.171	1.79394	−3.149	−1.260	−0.610	−0.167	0.364	0.819	1.295	1.165
46.255	1.79396	−3.097	−1.211	−0.590	−0.148	0.364	0.819	1.284	1.168
46.338	1.79392	−3.151	−1.238	−0.602	−0.163	0.363	0.803	1.291	1.186
46.421	1.79403	−3.157	−1.236	−0.618	−0.166	0.359	0.807	1.286	1.156
46.505	1.79395	−3.170	−1.240	−0.601	−0.148	0.377	0.819	1.280	1.149
46.588	1.79385	−3.138	−1.200	−0.577	−0.139	0.377	0.843	1.298	1.162
46.671	1.79407	−3.132	−1.209	−0.566	−0.157	0.406	0.844	1.317	1.189
46.755	1.79416	−3.142	−1.226	−0.600	−0.135	0.369	0.831	1.292	1.157
46.838	1.79422	−3.168	−1.229	−0.592	−0.157	0.364	0.816	1.282	1.160
46.921	1.79421	−3.130	−1.213	−0.605	−0.138	0.366	0.823	1.294	1.154
47.005	1.79412	−3.097	−1.222	−0.584	−0.163	0.377	0.822	1.274	1.191
47.088	1.79420	−3.125	−1.190	−0.598	−0.161	0.367	0.813	1.277	1.192
47.171	1.79412	−3.106	−1.211	−0.611	−0.163	0.343	0.823	1.306	1.164
47.255	1.79411	−3.103	−1.202	−0.592	−0.171	0.361	0.833	1.307	1.154
47.338	1.79399	−3.044	−1.192	−0.577	−0.150	0.359	0.834	1.287	1.195
47.421	1.79414	−3.088	−1.233	−0.589	−0.152	0.366	0.802	1.273	1.163
47.505	1.79407	−3.072	−1.208	−0.613	−0.156	0.348	0.824	1.274	1.196
47.588	1.79417	−3.046	−1.216	−0.602	−0.145	0.360	0.805	1.297	1.188
47.671	1.79394	−3.007	−1.202	−0.581	−0.134	0.350	0.832	1.289	1.179
47.755	1.79414	−3.029	−1.193	−0.601	−0.141	0.344	0.808	1.284	1.165
47.838	1.79419	−3.025	−1.202	−0.582	−0.132	0.358	0.807	1.275	1.161
47.921	1.79413	−2.972	−1.171	−0.583	−0.146	0.374	0.824	1.287	1.171
48.005	1.79406	−2.991	−1.171	−0.572	−0.158	0.365	0.809	1.277	1.170
48.088	1.79424	−3.000	−1.174	−0.564	−0.143	0.360	0.813	1.285	1.190
48.171	1.79422	−2.978	−1.176	−0.578	−0.146	0.357	0.807	1.259	1.155
48.255	1.79384	−2.910	−1.151	−0.558	−0.144	0.381	0.825	1.285	1.169
